# Intravascular imaging in coronary stent restenosis: Prevention, characterization, and management

**DOI:** 10.3389/fcvm.2022.843734

**Published:** 2022-08-09

**Authors:** Amr Abouelnour, Tommaso Gori

**Affiliations:** ^1^Zentrum für Kardiologie, Kardiologie I, Deutsches Zentrum für Herz und Kreislauf Forschung, University Medical Center Mainz, Mainz, Germany; ^2^Department of Cardiovascular Medicine, Cardiovascular Institute, Assiut University, Assiut, Egypt

**Keywords:** coronary, in-stent restenosis, intravascular imaging, intravascular ultrasound, optical coherence tomography, characteristics, management, prevention

## Abstract

Despite the introduction of drug-eluting stents to combat the neointimal hyperplasia that occurred after BMS implantation, in-stent restenosis is still encountered in a significant number of patients, particularly as increasingly complex lesions are tackled by percutaneous coronary intervention. Many biological and mechanical factors interplay to produce restenosis, some of which are avoidable. Intravascular imaging provided unique insights into various forms of stent-related mechanical issues that contribute to this phenomenon. From a practical perspective, intravascular imaging can therefore help to optimize the stenting procedure to avert these issues. Moreover, once the problem of restenosis eventuates, imaging can guide the management by tackling the underlying identified mechanism. Finally, it can be used to evaluate the re-intervention results. Nevertheless, with the emergence of different treatment options, more evidence is needed to define patient/lesion-specific characteristics that may help to tailor treatment selection in a way that improves clinical outcomes.

## Introduction

**FIGURE 1:**
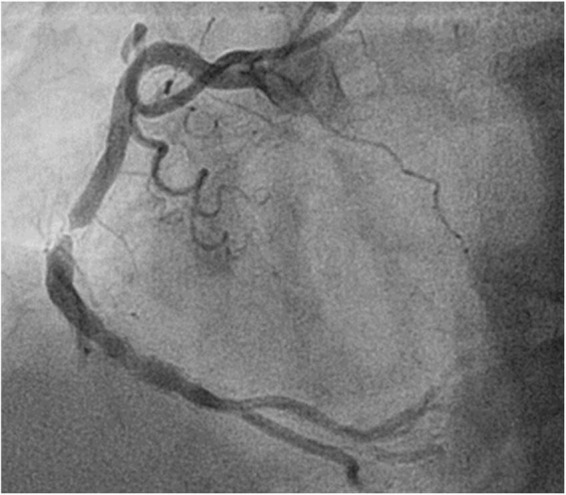
Angiographic appearance of an in-stent restenosis in segment 2 of the right coronary.

In spite of all the technological evolution of percutaneous coronary intervention (PCI) (Figure 1), in-stent restenosis (ISR) remains the commonest failure mechanism post-PCI, occurring in 3 to 20% of patients depending on patient’s and lesion characteristics and stent type (1). In the drug-eluting-stent (DES) era, the rate of ISR has decreased, but the absolute numbers actually increase due to the progression of PCI as a tool to treat increasingly complex coronary artery disease.

The traditional anatomic substrate of ISR is neointimal hyperplasia, resulting primarily from vascular smooth muscle cell proliferation. This classically occurred after bare metal-stent (BMS) implantation peaking early between 6 and 12 months. However, DES-ISR can peak years after implantation, the so-called “late catch-up” phenomenon (2). This is probably due to delayed healing and persistent inflammation, with the more frequent appearance of “neoatherosclerosis.”

Because various biological and mechanical mechanisms can contribute to DES ISR, e.g., drug resistance, hypersensitivity to the drug or polymer, etc. (3), the management of such challenging problem requires the identification of any underlying mechanical problems that can be rectified. Intravascular imaging with intravascular ultrasound (IVUS) or optical coherence tomography (OCT) allows this systematic investigation, to tailor interventions and tackle the underlying mechanism and to optimize the results of any necessary repeated interventions. In addition, intravascular imaging can call attention to potential new device-related issues. However, further evidence is still needed to prove that intravascular imaging-guided management of ISR improves clinical outcomes and/or prevents recurrences in this setting. From another standpoint, intravascular imaging has helped to clarify the relationship of certain lesion characteristics to the risk of ISR. For instance, IVUS has shown that although a well-developed collateral circulation (collateral flow index ≥0.25) is associated with more severe stenoses at baseline, it does not predict an increased risk of ISR (4).

Angiographic ISR is usually defined arbitrarily in binary terms as diameter stenosis of >50% in-stent or at its edges (5-mm segments adjacent to the stent) on coronary angiography (5). This provides simplicity but is also rooted in the physiologic significance of such degree of narrowing and has been shown to offer the best balance of sensitivity and specificity, compared to other more accurate but less accessible cutoffs, in terms of predicting clinically driven target lesion revascularization (TLR) (6, 7). However, because this is a 2-dimensional assessment, it is contingent on obtaining the worst-stenosis projection. Besides, it relies on the visual estimation of the operator that has to judge the ISR using the body of the stent, with a small margin of proximal and distal vessel. Even with the introduction of computer-assisted quantitative angiography, as a more objective clinical tool, limitations exist pertaining to the technology, technique, and analysis (8). Percentage diameter stenosis at follow-up coronary angiography and late luminal loss are also well-studied continuous-scale parameters used to describe the degree of restenosis (9). Late loss, as a measure of absolute renarrowing [ = minimum lumen diameter (MLD) immediately post-procedure − MLD at follow-up], was shown to be a generalizable and powerful endpoint across both BMS and DES, as well as across different stented vessel sizes. However, since the site of the MLD at implantation and that of MLD at follow-up do not need to be exactly the same, this measure does not reflect absolute neointimal proliferation. Percent diameter stenosis [ = (1 − (MLD/reference vessel diameter)) × 100], albeit another good measure to follow-up a given patient, is dependent on the reference vessel size, complicating its use as a comparator (10). In contrast, intravascular imaging allows a 3-dimensional cross-sectional assessment of the artery, permitting a direct visualization and quantification of the stent area, neointimal area, and luminal area (11), where restenosis is usually defined as a cross-sectional area re-narrowing >75% of the reference vessel segment (12). In addition, timely online quantitation is available rather than visual estimation. Earlier studies validated IVUS for *de novo* coronary lesions against stress myocardial perfusion single-photon emission computed tomography, as well as invasive coronary flow reserve, proposing criteria such as an IVUS minimum lumen area (MLA) <4.0 mm^2^ or corrected percent area stenosis ≥75% as the indicators of functional significance (13, 14). However, with the consolidation of myocardial fractional flow reserve (FFR) and other invasive physiological indices such as the instantaneous wave-free ratio (iFR) as the contemporary gold standard to assess the significance of coronary stenotic lesions, it became clear that many factors including ethnicity, vessel size, lesion location, the type of imaging used, etc., preclude the adoption of a universal cutoff by intracoronary imaging to intervene, and that intracoronary imaging can more readily provide thresholds for safe deferral of intervention (15).

The challenge of defining clinically relevant restenosis is compounded by the reality that the mere anatomic detection of a restenosis by angiography and intravascular imaging, especially if moderate, does not automatically signify a “clinical” or “functional” restenosis that can produce symptoms or objective evidence of ischemia. This uncoupling of anatomic and clinical restenosis might be attributed to the effect of other geometric aspects of the lesion (including its length) on flow, the status of endothelial function, collateral circulation, and the size of the subtended myocardial bed (9). Therefore, surrogate clinical endpoints such as TLR emerged, to capture a specific treatment’s ability to maintain long-term patency at the particular intervention site. The academic research consortium (ARC) criteria for TLR thus emphasize the clinical context in addition to anatomic luminal measurements, in the form of recurrent symptoms, objective signs of ischemia by non-invasive testing, or abnormal results of any invasive functional diagnostic test, unless the diameter stenosis is ≥70% (16, 17). Of note, this ARC consensus highlights the fact that early TLR events (<30 days after stenting) are unlikely to be due to ISR but are most likely a result of subacute stent thrombosis.

Intravascular imaging can guide the management of ISR through multiple stages. On the one hand, it can help to optimize the stenting procedure by predicting and avoiding ISR. On the other hand, once the ISR problem has set in, such modalities can help to identify the underlying mechanism. Finally, imaging can evaluate ISR treatment results.

## Prevention of restenosis

Intravascular ultrasound-guided BMS implantation was shown to modestly reduce restenosis and the need for repeat revascularization (18–20). However, the impact of IVUS-guided DES implantation on target vessel revascularization (TVR) was more controversial (21, 22). More recent studies and meta-analyses show that IVUS-guided PCI is associated with a significantly lower risk of TLR in all generations of DES, both in stable angina and in acute coronary syndromes (ACSs) (23–27). In complex PCI, earlier studies showed no impact of IVUS guidance on TLR (28), but later ones (or subgroup analyses thereof) confirmed that IVUS use reduces the risk of ischemia-driven TLR (29–32) and that such benefit is sustained on the longer term (33, 34).

Similarly, OCT guidance seemed to lower the 1-year risk of a composite that included TLR, only on unadjusted analyses (35). From another angle, when nearly 1,000 lesions in the same study were retrospectively analyzed, suboptimal stent deployment defined according to the presence of at least one of specific quantitative OCT criteria was associated with an increased risk of major adverse cardiac events (MACEs) including TLR. These criteria included in-stent MLA <4.5 mm^2^, distal dissection >200 μm, and distal or proximal reference lumen area (at stent edges) <4.5 mm^2^ in the presence of significant plaque (36). In another large observational study, where almost 90 thousand patients were analyzed (OCT used in over 1,100 patients, IVUS used in almost 11 thousand patients, and angiography alone used in slightly over 75 thousand patients), OCT-guided procedures were associated with a reduction in the prespecified primary endpoint of all-cause mortality at a median of nearly 5 years, but the study did not provide information about TLR on the long term, because the prespecified secondary composite endpoint was restricted to in-hospital events (37). From another perspective, OCT-guided PCI in patients with non-ST segment elevation ACS was shown to modestly improve the post-procedural fractional flow reserve (FFR), mostly by the optimization of stent expansion, when compared to fluoroscopy-guided PCI. In this study, post-PCI OCT revealed stent underexpansion in 42% of patients (38).

Interestingly, a prospective multicenter 1:1 randomized study has directly compared OCT to IVUS in patients undergoing PCI with a second-generation DES. The study successfully demonstrated that OCT-guided PCI was non-inferior to IVUS-guided PCI, regarding both angiographic in-stent and in-segment stenosis at 8 months, as well as ischemia-driven TLR at 12 months (39). Likewise, a meta-analysis incorporating that study among others, encompassing over 17 thousand patients, showed no difference in comparative efficacy between IVUS and OCT in terms of TLR, although OCT did not significantly lower the risk of TLR compared to angiography guidance (27).

This review provides an overview on the various forms of mechanical stent-related problems that can be detected by intravascular imaging and have been linked with varying degrees to ISR. Table 1 summarizes the relative strengths of IVUS and OCT for the detection of each of these problems.

**TABLE 1:** Relative strengths of IVUS and OCT for the detection of various underlying mechanisms of ISR.

Application/finding	Angiography	IVUS	OCT
Stent sizing	+	++	+
EEL/vessel wall visualization	−	++	+
Calcium pre-PCI	+ if severe	+ arc	+ arc and thickness/area
Peri-stent calcium	−	−	+
Acute stent malapposition	+	++	+++
Geographic miss	−	+	+
Edge dissection	+	++	+++
In-stent tissue prolapse	−	+	++
Stent fracture	+ (Stent-boost++)	+	++ (3D +++)
Longitudinal stent deformation	+	++	+++
Non-uniform strut distribution	−	+	+
Neointimal characterization	−	+	++
Multiple layers of stent	+	++	+++

+, feasible; ++, good; +++, very good.

### Stent underexpansion

**FIGURE 2:**
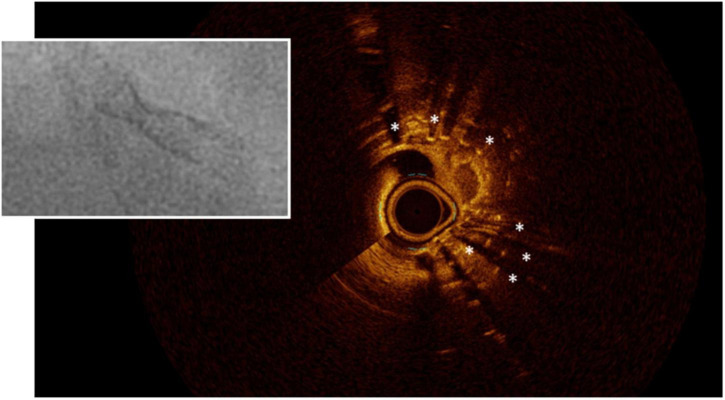
Stent underexpansion. OCT image of an incomplete expansion of a stent resulting in restenosis despite moderate neointima proliferation. The asterisks (*) mark stent struts. The small picture demonstrates the incomplete apposition at angiography.

The most frequent technically preventable mechanism of ISR in the DES era is stent underexpansion (40), often unrecognized at angiography (Figure 2). Post-PCI minimum stent area (MSA) is consistently the strongest predictor of ISR. In an early study of almost 500 lesions in 425 patients who underwent successful IVUS-guided stenting, based on the empiric criteria, an intrastent minimal lumen cross-sectional area ≥55% of the average reference vessel was the only criterion that was associated with a higher probability of freedom from ISR, independently from vessel size (41). However, more recent accumulating evidence suggested that the absolute stent expansion (MSA as an absolute measure) matters more than the relative expansion (MSA compared to a predefined reference area) in predicting the stent patency in the long term. In a study of over 1,500 patients [nearly 1,100 with paclitaxel-eluting stents (PESs), and nearly 500 with BMS], post-PCI MSA was the independent predictor of subsequent ISR at 9 months, with an optimal threshold of 5.7 mm^2^ for PES (42). Similarly, for sirolimus-eluting stents (SESs), post-procedural final MSA by IVUS was one of the only two independent predictors of angiographic restenosis; the other predictor being the stent length. In that study, the final MSA cutoff that best predicted ISR was 5.5 mm^2^ (43). Similar data have been shown for the second-generation DES, where a study of almost 1,000 lesions identified the post-stenting MSA as the only independent predictor of angiographic ISR in zotarolimus-eluting stents (ZESs) and in everolimus-eluting stents (EESs). The best MSA cutoff value was 5.5 mm^2^ for the prediction of SES restenosis, 5.3 mm^2^ for ZES ISR, and 5.4 mm^2^ for EES (44). Most recently, this was challenged by an IVUS substudy of the ADAPT-DES registry (Assessment of Dual Antiplatelet Therapy with Drug-Eluting Stents), where ten different stent expansion indices were compared for their association with a primary endpoint of lesion-specific 2-year clinically driven TLR or definite stent thrombosis. Interestingly, only MSA/vessel area at the MSA site (best cutoff was 38.9%) was independently associated with the study endpoint, after adjusting for morphologic and procedural parameters. In other words, stent/vessel area at the MSA site was superior to absolute MSA (and other expansion indices) in predicting the study endpoint, driven mainly by the difference in TLR rather than stent thrombosis (45).

Whereas the studies mentioned above investigated non-left main lesions, Kang et al. showed that for unprotected left main (LM) stenting, ISR was more frequent in lesions with underexpansion of at least one segment, with a significantly lower event-free survival rate than in lesions with no underexpansion. The MSA cutoffs that best predicted ISR on a segmental basis were 5.0 mm^2^ for ostial left circumflex (LCX), 6.3 mm^2^ for ostial left anterior descending (LAD), 7.2 mm^2^ for the polygon of confluence (POC), and 8.2 mm^2^ for the LM above the POC (46).

Likewise, in chronic total occlusions (CTO) that are successfully recanalized, IVUS has shown that a smaller MSA is a major predictor of angiographic ISR (47). Additionally, similar to patients with stable coronary artery disease, a smaller MSA after primary PCI was shown to be an independent predictor of angiographic restenosis (48).

Concurring with the intravascular ultrasound (IVUS) findings, post-stenting optical coherence tomography (OCT) has shown that a small MSA (defined as <5.0 mm^2^ for DES) is an independent predictor of 1-year device-oriented clinical endpoints (49). It is important to note, however, that these thresholds do not define optimal stenting, such that a larger MSA is still associated with less ISR, until an MSA of about 8 mm^2^, where a plateau is reached (50).

Interestingly, when compared to phantom models, OCT was more accurate and reproducible in the assessment of coronary luminal dimensions than IVUS which overestimated those dimensions and was less reproducible (51). Earlier studies, however, suggested that OCT guidance would yield smaller stent expansion and more residual reference segment stenosis than IVUS guidance, because of the greater ability of IVUS to visualize the vessel border compared to OCT, both before and after intervention. This would potentially lead to more stent restenosis (52). Nevertheless, when OCT-guided stenting was compared to IVUS-guided stenting in the ILUMIEN (optical coherence tomography compared with intravascular ultrasound and with angiography to guide coronary stent implantation) II study, both approaches resulted in a comparable degree of stent expansion, defined as MSA divided by the mean of the proximal and distal reference lumen areas (53).

Regarding the relative stent expansion, the EAPCI consensus views a cutoff of >80% for the MSA relative to average (proximal and distal) reference lumen area, as a reasonable and realistic target to employ in clinical practice, taking into consideration that more stringent targets such as >90% were frequently not achieved in the respective clinical trials (54).

Stent underexpansion could be due to stent underdeployment (the use of low deployment pressures), stent undersizing, or heavily calcified lesions that preclude adequate stent expansion despite high deployment and/or post-dilation pressures. It is important therefore to recognize target lesion calcium, so as to consider pre-stenting calcium modification techniques, e.g., rotational, or orbital atherectomy, cutting, or scoring balloons. Intravascular imaging is more sensitive for calcium detection than angiography. In a study of 1,155 native vessel target lesions in stable patients, IVUS detected calcium in 73% whereas angiography detected calcium in only 40% (55). This is in line with the results of other studies, which showed a good sensitivity and specificity of IVUS to detect intralesional calcium compared to pathology. In one study that examined the ability of IVUS to accurately depict circumferential calcified lesions on autopsy arterial segments, IVUS had a sensitivity of 89% and a specificity of 97% (56). In another study of 50 coronary vessel segments, IVUS was compared to corresponding histologic sections and had an overall sensitivity of 90% and a specificity of 100% for the detection of dense, coherent calcium, even though it had a much lower sensitivity (64%) for visualizing microcalcification (57).

**FIGURE 3:**
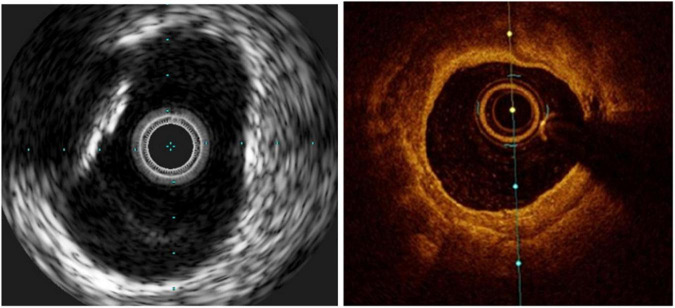
Calcific lesions at intravascular ultrasound **(Left)** and optical coherence tomography **(Right)**. The limitation of IVUS in this case is that the lesion projects a shadow that does not allow measuring its depth.

**FIGURE 4:**
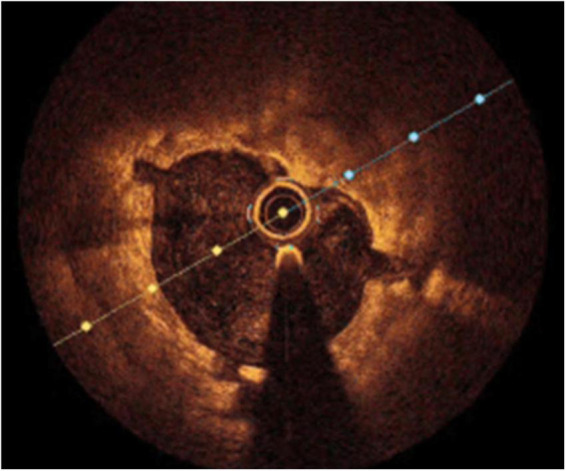
Cracks in the subintimal calcium after coronary lithotripsy.

While it is difficult to evaluate calcium thickness or area with IVUS because its surface reflects ultrasound waves almost entirely, OCT can penetrate through calcium, so that its thickness and area can be evaluated (Figure 3). This was shown to be relevant, although to a lesser degree than the arc of calcium, so that it affects the minimal stent diameter achieved (58). In another study, a thinner calcium thickness after rotational atherectomy (optimal threshold was 0.67 mm) predicted the formation of cracks after balloon angioplasty which in turn permitted a larger lumen gain and a greater stent cross-sectional area (59). An OCT-based calcium scoring system has been proposed, whereby a maximum angle of calcium >180°, together with a maximal thickness >0.5 mm, and length >5 mm, predicted stent underexpansion (based on the smallest stent area divided by the average of proximal and distal reference luminal areas) with a slightly better ability than the angiographic detection of severe calcium (60). On the other hand, it has been demonstrated that the detection of calcium fractures by OCT (Figure 4) confirms adequate modification of heavily calcified culprit lesions before stenting and resulted in a greater MSA, and stent expansion immediately post-PCI, as well as smaller percent diameter stenosis, less frequent binary restenosis, and less ischemic-driven TLR, at 10-month follow-up (61).

### Stent undersizing

There is no clear-cut evidence to support routine use of intracoronary imaging for stent sizing. However, there is some evidence to support intracoronary imaging guidance in long lesions and CTOs (62–64), although the greater CTO success achieved in the imaging-guided group in terms of, e.g., a greater MSA (at least in part due to bigger implanted stent diameters), did not always translate into better clinical outcomes, with the exception of less stent thrombosis and less TLR in longer lesions (65, 66). In addition, the EAPCI expert consensus recommends its use in LM lesions, patients with ACS, or other complex lesion morphologies (67). Nevertheless, there seems to be less benefit in simple lesions or patients with stable clinical presentation (54, 68).

Imaging-guided stent sizing is based on either the external elastic lamina (EEL) diameters of the distal reference, usually rounded down by ≥0.5 mm or alternatively, reference lumen diameters can be used, rounded up by 0.5 mm. Particularly in smaller or very calcific arteries, or in the setting of diffusely diseased vessels (including chronic total occlusions) or acute coronary syndromes, imaging-guided sizing is often larger than the angiographic reference diameters.

**FIGURE 5:**
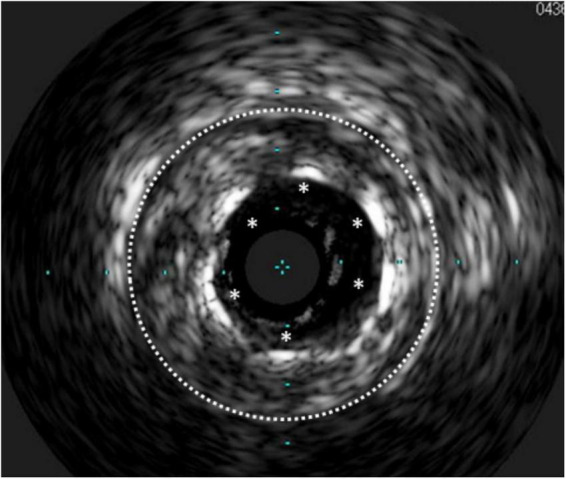
Stent undersizing demonstrated at IVUS. The white dotted line marks the lamina externa, where the stent struts (*) should lie.

**FIGURE 6:**
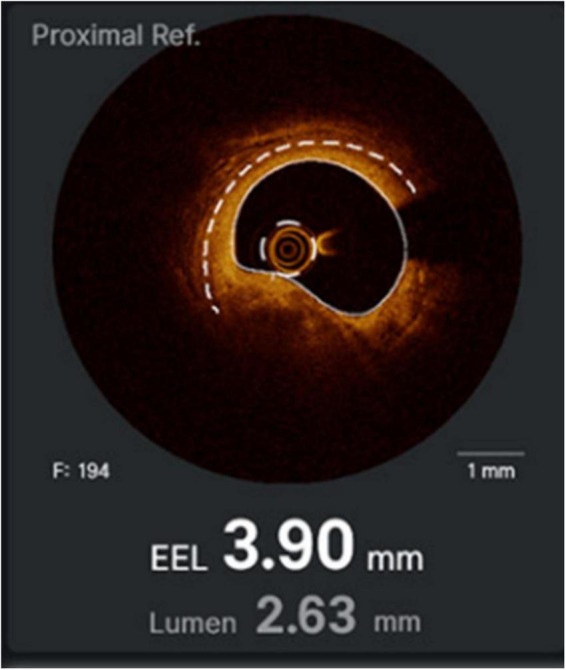
Artificial intelligence-driven stent/vessel sizing.

For its superior capacity to distinguish the EEL, IVUS is considered to be the gold standard method for guiding stent sizing (Figure 5). Because of the limited tissue penetration of OCT (1–2 mm) compared to IVUS (5–6 mm), it is often not able to visualize the EEL at the lesion site. The introduction of artificial intelligence-based methods in the latest iteration of the OCT software by the company Abbott vascular, however, significantly streamlines these processes (Figure 6). Therefore, an algorithm was proposed by the ILUMIEN III and IV studies (54, 69), where the EEL diameter was used if the EEL circumference was visible for ≥180°. In such case, the proximal and distal reference mean EEL diameters were measured, and the smaller of these diameters rounded down to the nearest 0.25 mm was used. In case the EEL cannot be seen ≥180°, the stent diameter was determined as 100% of the lumen diameter. Compared to the respective reference, a final lumen area ≥90% was considered acceptable. Using this algorithm, OCT-guided PCI was non-inferior to operator-directed IVUS guidance in terms of the post-PCI MSA, with no difference in procedural MACE up to 1 year (54, 68). Because ILUMIEN III was underpowered to detect differences in clinical outcomes, another adequately powered ongoing trial, ILUMIEN IV trial (NCT03507777), is geared to rigorously test this aspect (69).

From a practical perspective, the EAPCI consensus on the clinical use of intracoronary imaging highlights the distal lumen reference-based sizing as a safe and straightforward approach, which can be followed by an optimization of the mid and proximal stent segments. The mean distal lumen diameter with up rounding of the stent diameter by 0–0.25 mm may be used, or alternatively, the mean EEL (if adequately visualized) with down rounding to the nearest 0.25 mm stent size can be used (67).

### Geographic miss

Longitudinal geographic miss (GM) refers to an injured or diseased vessel segment not covered by the stent. Both IVUS and OCT provide valuable information to determine an appropriate (relatively plaque-free) landing zone for stent implantation, to avoid GM and have adequate lesion coverage.

In a study of over 1,500 patients with SES implanted, GM had a four-fold increase in the incidence of edge restenosis, with a significantly higher rate of TVR at 1 year, after adjusting for clinical and anatomic factors (70). In another smaller retrospective study of 167 SES, a larger reference percentage of plaque area at baseline IVUS was among the factors associated with edge restenosis (71). Similarly, residual edge plaque burden was the only independent predictor of angiographic stent edge restenosis at 9 months after PES implantation (72). Another IVUS study of newer generation DESs in almost 1,000 lesions concluded that edge restenosis was predicted by post-stenting reference segment plaque burden and reference segment MLA. The predictive cutoff of the reference plaque burden was 54.5% overall (56.3% for endeavor ZES, 57.3% for resolute ZES, and 54.2% for EES) (73).

Comparable results were obtained by OCT in a retrospective study of 319 patients with EES implantation. The independent predictors of binary angiographic stent edge restenosis at 9 to 12 months were lipidic plaque in the stent edge segment (optimal cutoff was an arc ≥185) and MLA (optimal cutoff was 4.1 mm^2^) (74).

Interestingly, in a prospective single-center randomized study of 200 patients, comparing OCT-guided PCI with vs. without co-registration, there was no significant difference in GM, even though co-registration enabled more precise stent location. There was also a trend for less severe edge dissection with co-registration, which did not reach statistical significance (75).

### Edge dissection

**FIGURE 7:**
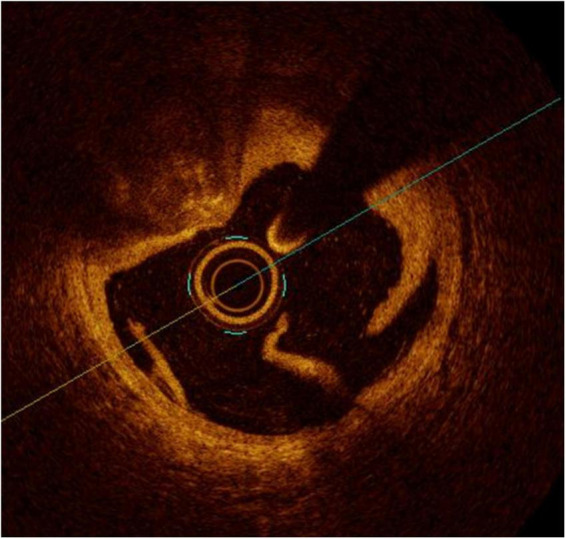
Edge dissection.

Optical coherence tomography has a greater sensitivity to detect post-procedural stent edge dissections than IVUS (Figure 7), attributable to its greater resolution. This is confirmed, for example, by data from ILUMIEN III, where untreated edge dissections as detected by OCT were more frequently present after IVUS-guided and angiography-guided PCI than after OCT-guided PCI. Not only untreated major dissections (≥60° in circumference and/or ≥3 mm in length) and medial dissections were more common after IVUS-guided PCI than after OCT-guided PCI, but indeed, OCT detected nearly 25% of total dissections and 15% of major dissections that were overlooked by IVUS (54). This agrees with the data from other smaller-scale studies (51). However, these findings had no impact on stent-oriented outcomes (68).

On the other hand, although it has previously been shown in a porcine model that the degree of arterial injury is strongly correlated with the magnitude of restenotic response (76), and one group has shown that IVUS-detected edge dissections were related to more restenosis with subsequent TLR (77), Radu et al. demonstrated, however, that non-flow limiting edge dissections identified by OCT at baseline completely healed on serial follow-up at 1 year, except for the longest flaps (2.81 and 2.42 mm), which were only partially healed. In all cases, however, this did not produce restenosis as assessed by OCT at 1-year follow-up, nor did it result in MACE within that year. Moreover, the two cases with persistent/partially healed dissections had no MACE up to 3 years of follow-up (78). This aligns with a multitude of other studies that employed angiographic (71, 73, 74, 79), IVUS (80), and OCT follow-up (81–84). Similarly, in a study of over 1,000 stents implanted to treat 900 lesions (including both BMS and DES), stent edge dissection detected by OCT had no impact on device-oriented clinical endpoints at 1 year of follow-up (49).

Of note, other groups related certain OCT-derived characteristics of the edge dissection to MACE, e.g., dissection flap thickness ≥200 μm (85), or 310 μm (86), or dissection length >3.55 mm (87), but none of these groups demonstrated an association specifically with edge restenosis.

### In-stent tissue protrusion/prolapse

**FIGURE 8:**
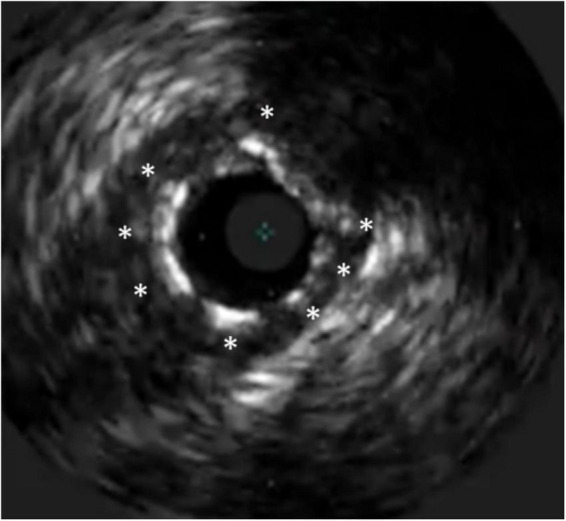
Tissue prolapse after PCI of a chronic total occlusion. The stent struts are marked with *, at 1 o’clock a prolapse of a calcific plaque can be seen.

Optical coherence tomography seems to be more sensitive than IVUS for intrastent tissue protrusion detection (51) (Figure 8). However, the impact of tissue protrusion/prolapse (TP) on restenosis and TLR is controversial, with several studies failing to establish a significant relationship

An early study of 407 native coronary lesions, where post-intervention IVUS was done, failed to show an association of minor plaque prolapse (detected in nearly quarter of the lesions) and 6-month angiographic restenosis (88). In another serial IVUS study of 205 lesions in patients with diabetes, plaque prolapse was not associated with increased neointimal proliferation or angiographic restenosis (89).

From the OCT perspective, irregular in-stent TP independently predicted 1-year device-oriented clinical endpoints, primarily driven by TLR that was not for stent thrombosis. This is probably because irregular tissue protrusion represents a moderate to severe vessel injury with a high likelihood of medial disruption and lipid core penetration resulting in restenosis (49). However, in another serial OCT and IVUS study, although the lumen area in lesions with TP significantly decreased due to neointimal proliferation at follow-up (with greater late lumen area loss in IVUS-detected than in OCT-only-detected TP), no impact on clinically relevant restenosis was demonstrated (81).

Interestingly, in a prespecified substudy of the ADAPT-DES, TP detected by IVUS was associated with less clinically driven TLR at 2 years, in part because of greater stent expansion in these lesions which was presumably among the causes of TP, and because greater stent expansion counterbalanced the impact of TP to maintain a good acute result in terms of luminal dimensions (90).

### Acute stent malapposition

**FIGURE 9:**
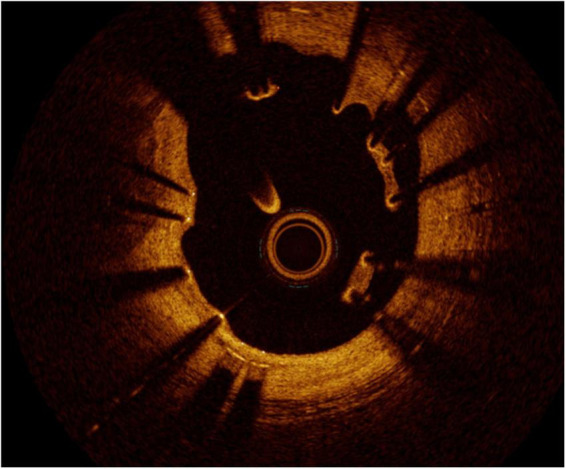
Malapposition. Although not being a direct cause of restenosis, the flow disturbance caused by the malapposed struts may cause neointima proliferation distal to the site.

Optical coherence tomography has a greater ability to detect acute stent malapposition (ASM) than IVUS (Figure 9). For instance, when the same lesions were evaluated post-PCI with frequency-domain OCT and IVUS, incomplete stent apposition was detected in more lesions by OCT than by IVUS (39 vs. 14%) (51). Similarly, in the ILUMIEN III trial, OCT was significantly more sensitive than IVUS at detecting malapposition. Moreover, OCT procedural guidance led to fewer malappositions than did IVUS guidance (54).

The clinical outcome of ASM yet is uncertain, i.e., no clear connection exists between ASM (in the absence of underexpansion) and subsequent TLR. This is probably because ASM may subsequently resolve. In a serial OCT study of 66 stents (including EES, ZES, and BMS), 71.5% of the ASM segments were completely integrated into the vessel wall at 6-month follow-up. The maximum ASM distance per strut (or ASM volume) was the only predictor for the percentage of malapposed struts at follow-up, so that a maximum ASM distance <270 μm was grossly covered and spontaneously reapposed in 100% of cases at follow-up, whereas distances ≥850 μm resulted in persistent ASM and delayed coverage in 100% of cases (91). The same concept was demonstrated in another serial OCT study of 77 patients, which showed spontaneous resolution of ASM at 8- to 12-month follow-up, which occurred to a greater extent in EES than in SES. The best cutoff of the ASM acute distance for predicting late persistent malapposition at follow-up was >355 μm for EES and >285 μm for SES (92). A third OCT study with a longer follow-up of 2 years has shown a very close cutoff endoluminal distance for the resolution of ASM in cobalt chromium EES of 359 μm (93).

Consequently, in one study, for example, of over 350 lesions, where patients received post-stenting OCT, ASM was observed in 62% of lesions, and yet, there was no difference in clinical events including TLR between patients with and without stent malapposition (94). In another retrospective analysis of post-procedural OCT findings in 864 patients, variable grades of ASM were detected in 72.3% of stents, but had no relation to ISR, nor to TLR (95). Similarly, in acute coronary syndrome patients, post-procedural OCT-detected ASM was not associated with device-oriented events including TLR (96). On the other hand, in an integrated analysis of IVUS substudies of multiple trials, where 1,580 patients were evaluated (with either PES or BMS implanted), stent malapposition had no impact on the rates of restenosis nor MACE including TLR at 9 months (97). Furthermore, in the IVUS substudy of ADAPT-DES trial, ASM did not influence clinically driven TLR at 2-year follow-up (98).

### Stent fracture

A stent fracture undermines the scaffolding at its site as well as the local drug delivery in the case of a DES. This is coupled with mechanical irritation of the vessel by the fractured struts. Moreover, evidence has been shown by IVUS for recoil of the edges of the struts just proximal and distal to the fracture site (99). Therefore, stent fractures associate with higher rates of ISR and TLR (100–102). Intravascular imaging can help to identify the cases of stent fracture overlooked on angiography, in the context of ISR (103, 104), particularly with overlapping of the proximal and distal fragments (99). Sometimes, stent fracture is followed by longitudinal overlapping that shows up as a single arc of double layers of stent struts in the same circumference on consecutive frames in the middle of a single stent (99). Three-dimensional OCT provides further help in challenging cases such as single strut fractures (105, 106). Nevertheless, stent-boost technology and multidetector computed tomography can frequently make the diagnosis obviating the need for dedicated intracoronary imaging (107, 108).

**FIGURE 10:**
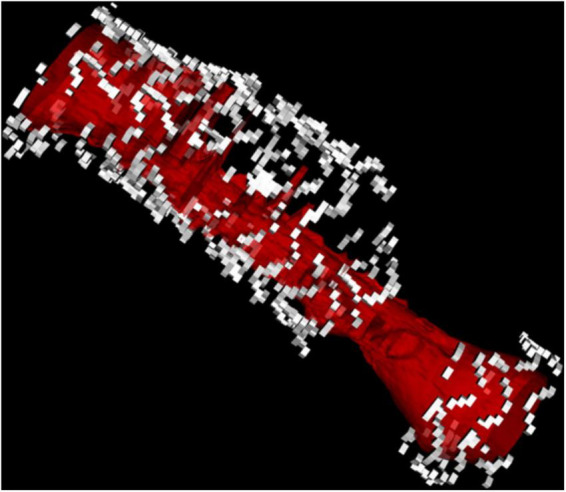
3D reconstruction of a stent fracture and gap associated with ISR (the lumen is depicted in red).

In a recent case series, our group reported that fracture, classified in four different patterns, typically results in a focal ISR at the fracture point (Figure 10) (109). In that study, we proposed an OCT-based classification in different patterns of increased severity (from single stacked struts to rupture and gap), and indeed, more complex fracture patterns were more common in the presence of device failure than in incidentally discovered fractures. In the same study, we also reported an association of stent fractures with stent eccentricity and asymmetry, which theoretically are among aspects that intracoronary imaging can help to optimize. Once a fracture is diagnosed, it is yet to be investigated in further studies how such imaging-based classification scheme would inform the management.

### Longitudinal stent deformation

It is uncertain whether longitudinal stent deformation (LSD) relates to outcome including ISR. In a pooled analysis of patients treated with ZES and EES, rates of target lesion failure were numerically but not significantly higher in lesions with quantitative coronary angiography (QCA)-based LSD (110). However, in another study of EES-treated lesions, it was shown that LSD with resulting overlap leads to an excessive neointimal hyperplasia (NIH), ISR, and TLR (99). The mechanism is unclear but is probably non-uniform drug distribution.

### Non-uniform strut distribution

Takebayashi et al. have demonstrated in patients with SES implanted stents, that non-uniform strut distribution represented by a larger maximum interstrut angle on IVUS analysis independently predicted NIH formation and subsequent restenosis (111).

## Characterization of restenosis

Mehran et al. originally proposed an angiographic classification for ISR (classes I to IV) that predicted more TLR with increasing ISR class (112). Although the classification was based on the geographic position of ISR on angiography in relation to the previously implanted stent, its accuracy was verified in the same study by IVUS. Despite that, the classification has several limitations; for instance, it was developed in the BMS era just as DES was dawning. Consequently, in RIBS-II trial, for example, which compared SES with balloon angioplasty as a treatment for ISR, the Mehran classification was unable to predict late loss in the SES group (113). On the other hand, such classification does not really address the possible underlying causes for ISR, nor does it describe the nature of the restenotic tissue itself. As a result, it would not be able to prescribe management pathways given the plethora of newly developed interventional tools available to tackle ISR.

Intracoronary imaging has greater sensitivity to detect and characterize ISR (114). In an IVUS study that investigated the patterns of ISR among different stent generations, BMS restenosis presented later with more NIH compared to DES (including both first- and second-generation DES). Additionally, the total stent length was longer in DES ISR, and the stent cross-sectional area at the site of the minimal luminal area (MLA) was smaller, compared to BMS ISR. Regarding the underlying mechanism, stent fracture was seen only in DES, but stent malapposition was seen equally across all stent generations (115). In a prospective multicenter registry conducted in Nordic and Baltic countries, IVUS showed that stent underexpansion was more common in DES ISR than in BMS ISR, and that DES more frequently had focal ISR compared to BMS, with less intimal hyperplasia (116).

**FIGURE 11:**
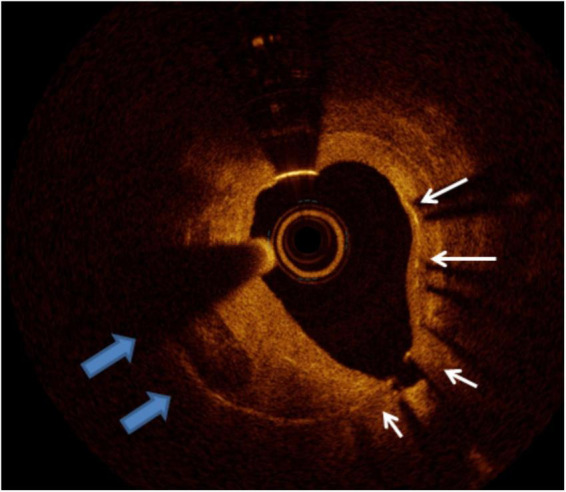
Calcific neointima. The white arrows mark the stent struts, the left quadrant arc occupied by neointima presenting calcific neoatherosclerosis. The remaining homogeneous neointima is compatible with fibrous tissue.

**FIGURE 12:**
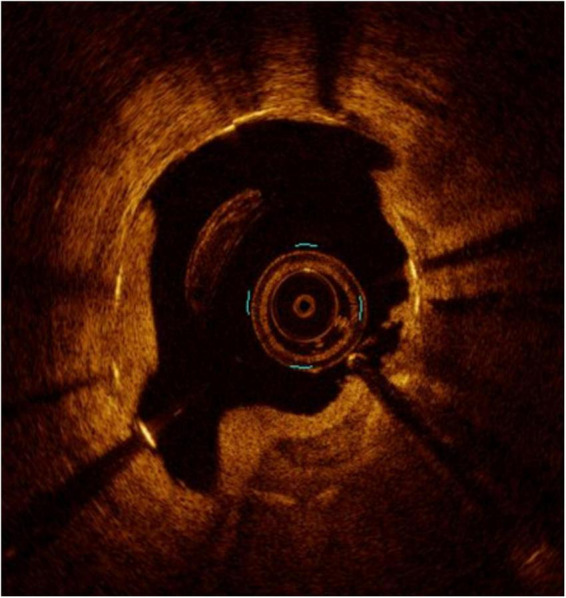
Neoatherosclerosis.

**FIGURE 13:**
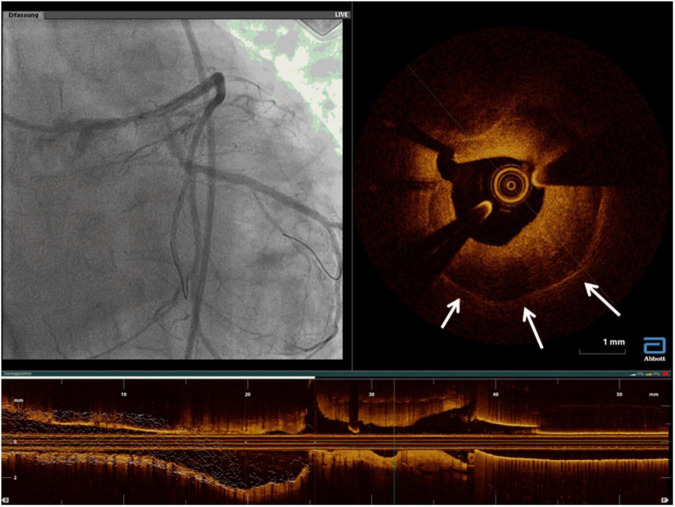
A case of rapidly growing in-stent restenosis of the left main (3 months from previous angiography). The white arrows mark areas of peri-strut low-intensity compatible with inflammatory processes and with a rapid progression of disease.

Because of a higher resolution, OCT has permitted more detailed characterization of the underlying etiology of ISR. Furthermore, it highlighted the morphologic difference between DES-ISR and BMS-ISR (117). In other words, OCT allowed better characterization of the neointimal tissue type, including identification of in-stent neoatherosclerosis, which could potentially guide therapy. In BMS-ISR, the typical pattern is a homogeneous high-signal tissue band, which is a characteristic of neointimal hyperplasia, with high smooth muscle cell content (Figure 11). In contrast, DES-ISR is typically characterized by attenuated, layered, heterogeneous tissue, which may represent proteoglycan-rich neointimal tissue, or neoatherosclerotic plaque (Figure 12) (118). Regions of the so-called peri-strut low-intensity (PSLIA) have been associated with accelerated restenosis due to inflammation, proteoglycan accumulation, and edema (Figure 13). Therefore, whereas neointimal formation peaks at about 6 months after BMS implantation, neointimal formation after DES implantation is a dynamic process that could creep out to even 5 years (118). In-stent neoatherosclerosis can also cause DES failure, through intimal rupture and thrombus formation, which usually presents with an ACS rather than stable angina (26). Of note, some studies suggest that stent age (i.e., longer implant duration) rather than stent type is the strongest and most consistent predictor of neoatherosclerosis (119).

Similar findings have been elicited by integrated backscatter (IB) ultrasound which revealed more low-IB tissue in the neointima of late restenosis (detected at ≥13 months after stent implantation) than in that of early restenosis, suggesting neoatherosclerosis as one of the mechanisms of late ISR (120). Furthermore, in patients with DES ISR, it seems that the underlying mechanism can impact the restenotic tissue composition, e.g., stent fracture has been shown by IB-IVUS to be associated with larger lipid volume within the neointima, indicating a contribution to the development of neoatherosclerosis (121).

A classification of ISR has been proposed based on the qualitative OCT assessment of a small mixed sample of BMS and different generations of DES, where restenotic tissue was described as “layered,” “homogeneous,” or “heterogeneous.” These patterns were not validated against histologic data, and no insights were provided as to the clinical and prognostic value (122). Recently, Yamamoto et al. proposed a novel classification of ISR after DES implantation, based on OCT imaging, that should be more accommodating of the different ISR tissues encountered. In total, six patterns were proposed (Supplementary Figure 1): Type I is a “homogeneous high-intensity tissue” pattern, representing neointimal tissue composed of smooth muscle cells in a proteoglycan and collagen-rich matrix; type II is a “heterogeneous tissue with signal attenuation” pattern and was the most frequent pattern reported, suggestive of delayed arterial healing and/or the presence of an organized thrombus; type III is a “speckled heterogeneous tissue” pattern, indicating organized thrombus and fibrinoids with smooth muscle cells poorly and focally distributed, probably an early phase of type II; type IV is a “heterogeneous tissue containing poorly delineated region with invisible struts,” comprising atheromatous tissue, including large fibroatheroma or a large amount of foam cell accumulation within the neointima; type V is a “heterogeneous tissue containing sharply delineated low-intensity region,” representing dense calcified plates; and type VI is a “bright protruding tissue with an irregular surface,” representing calcified nodules. Because this classification has the potential to differentiate lipidic atherosclerotic neointima and calcified neointima from other neointimal tissue, it may help in guiding the treatment strategy. Although in high-lipid-content neoatherosclerosis, minimal lesion preparation would be preferable to reduce no-reflow and periprocedural infarction, in calcified neointima aggressive preparation is necessary to permit adequate stent expansion (123). Importantly, Imanaka et al. validated OCT patterns of neointimal tissue following DES implantation against histopathology and showed that the heterogeneous pattern with invisible strut on OCT identifies the presence of lipid atherosclerotic tissue within neointima (124). Systematic prospective studies are emerging to define the clinical implication of these morphological patterns on prognosis and management.

Another group used OCT gray-scale signal intensity to provide a quantitative neointimal analysis and expectedly found significantly different values among the different neointimal prototypes (homogeneous, non-homogeneous, and neoatherosclerosis), although different patterns coexisted in a significant proportion of ISR lesions. However, no correlation to the gold standard of histology was offered, nor is the impact on clinical and angiographic outcomes clear (125).

## Evaluating restenosis by intravascular imaging vs. non-invasive imaging

Non-invasive imaging in the setting of ISR was primarily investigated as a method of surveillance/screening in lesions with a reported relatively high rate of ISR, e.g., LM PCI, to avoid resubjecting the patient to invasive coronary angiography for that purpose.

Van Mieghem et al. showed in a population undergoing LM stenting that multisclice computed tomography (CT) by a 16-slice scanner had good agreement for measuring the mean stent cross-sectional area but did not report the Bland-Altman results for the MLA (126). With the introduction of 64-slice multidetector CT (MDCT), Andreini et al. examined the agreement between luminal area measurements on CT to those on IVUS in a group of 24 patients. MDCT systematically underestimated the MLA compared to IVUS, with wide limits of agreement (127). Later, Veselka et al. have demonstrated that dual-source computed tomography (by measuring the MLA) can exclude in-segment restenosis after LM bifurcation stenting with a negative predictive value (NPV) of 97%, using IVUS-measured MLA as the gold standard (128).

Another very appealing application is the possibility of using CT to efficiently diagnose stent fracture, which as described is one of the underlying mechanisms for ISR (107, 108, 129, 130).

On the other hand, Kang et al. compared angiographic and IVUS assessment of ISR severity (LM and multivessel ISR were not included) to single-photon emission computed tomography (SPECT) as a measure of functional significance of the lesion. The overall diagnostic accuracy of IVUS-derived parameters to predict a positive SPECT was only 70% similar to that of angiography in the same study (an MLA cutoff ≤2.1 mm^2^, which performed best among other parameters, had a positive predictive value (PPV) of only 62%, and a NPV of only 77%) (131). This can be attributed on the one hand to the inherent limitations of SPECT, e.g., artifacts, spatial resolution, the confounding effect of microcirculation, etc., with the potential of false positives and negatives, and on the other hand, to the multitude of factors that influence the IVUS assessment, e.g., the need for different cutoff MLA values for different lesion locations, varying vessel diameters, and different ethnicities or body habitus (15).

## Management of restenosis

**FIGURE 14:**
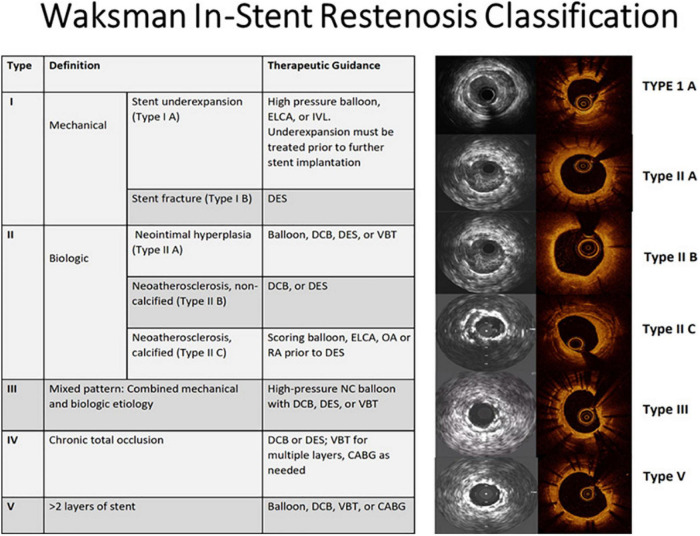
Imaging-guided treatment of ISR. ELCA, excimer laser coronary atherectomy; IVL, intravascular lithotripsy; DES, drug-eluting stent; DCB, drug-coated balloon; VBT, vascular brachytherapy; OA, orbital atherectomy; RA, rotational atherectomy; NC, non-compliant; CABG, coronary artery bypass graft. Reproduced with permission from Shlofmitz et al. (136).

The initial step in ISR management is identifying the underlying cause which in turn dictates the treatment strategy (Figure 14). Intracoronary imaging can help to differentiate between a mechanical cause and a biological cause and discriminate between various mechanical causes. That said, clinical evidence supporting a clear advantage for routine intravascular imaging in ISR is still lacking. In other words, there is a lack of evidence on the use of individualized therapeutic strategies to target specific underlying ISR substrates detected by intravascular imaging, and whether this individualized approach improves clinical outcomes. Therefore, the 2018 ESC guidelines on myocardial revascularization give a class IIa C recommendation for the performance of IVUS and/or OCT to detect stent-related mechanical problems leading to restenosis (132). The use of intracoronary imaging is also recommended by a recent expert consensus of the EAPCI on the management of myocardial revascularization failure (133). Along the same lines, the 2021 ACC/AHA/SCAI guideline for coronary artery revascularization provides a class 2a C-LD recommendation for the use of IVUS or OCT to determine the mechanism of stent failure (134). Early on it seemed as if the only independent predictor of late clinical outcome after re-intervention for ISR was the final MLA obtained, regardless of the means used to achieve it (135). An optimal treatment algorithm is discussed in detail in two recent excellent reviews by Waksman et al. (114, 136). The algorithm overall is in line with the corroborating evidence available in different situations as presented below.

From a practical standpoint, in cases of stent undersizing or underexpansion (Waksman type I A), identifying the tissue composition can guide optimal device choice. Soft tissue will probably respond to treatment with high-pressure balloon inflation, whereas calcific lesions might require other devices including rotational atherectomy, intravascular lithotripsy (IVL), or excimer laser coronary angioplasty (ECLA). Similar calcium modification prior to DES would be needed in the case of calcific neoatherosclerosis (Waksman type II C) (136).

In retrospect, peri-stent calcium-related stent underexpansion can be detected by both IVUS and OCT, but OCT can better evaluate the thickness of calcium. It is often the result of the underutilization of calcium modification tools before stenting such as rotational or orbital atherectomy, or ECLA. Possible management options for this problem include rotational atherectomy, ECLA, and most recently IVL.

The off-label use of rotational atherectomy “stent-ablation” was reported in a small-sized retrospective non-randomized study to achieve a lower but acceptable rate of procedural success, and a similar rate of procedural complications compared to excimer laser with contrast media injection (if certain intraprocedural precautions are observed to avoid dissections and burr entrapment) (137). In another observational single-center study of 11 patients, stent-ablation had a high rate of procedural success (all but one patient), with MACE occurring in only one patient at a median follow-up of 26 months (138). In this situation, rotational atherectomy is capable of ablating metallic stent struts, but it is the fragmentation of any underlying calcific or fibrotic tissue surrounding the unexpanded stent that is thought to facilitate the following expansion of the stent (137). A third group studied 12 patients in a prospective registry. Despite excellent procedural results, MACE occurred in 50% of patients at 6-month follow-up, which could be attributed at least in part to the modest outcomes and the higher intrinsic risk of such patients. Consequently, the authors emphasized the importance of ample lesion preparation upfront, to limit such bailout situations (139).

Several authors reported individual case reports of using ECLA to expand an undilatable stent, but the ELLEMENT registry was the first large prospective, multicenter observational registry (*n* = 28) to evaluate contrast-enhanced laser therapy to modify stented plaques resistant to high-pressure balloon expansion. Procedural success was achieved in 27 patients (96.4%), with MACE occurring only in two patients at 6-month follow-up (one death and one TLR) (140).

Recently, studies reported that OCT-diagnosed calcium can be effectively treated with IVL to induce calcium fracture and thus achieve larger final MLA and MSA independently of the eccentricity of the lesion (141–144). In the disrupt CAD III OCT substudy, the mechanism of calcium modification appeared to be circumferential and longitudinal calcium fractures (identified in almost 70% of the lesions), but the subsequent stent expansion and MSA were similar regardless of calcium fracture identification by OCT (144). The use of this device in ISR has been reported by several groups but is currently off-label (145–149). For instance, in a recent retrospective single-center analysis of six patients (two with calcium-mediated stent underexpansion, two with calcified neointima, and two with both), IVL with subsequent high-pressure balloon dilation (followed by DES implantation or drug-coated balloon (DCB) deployment) was feasible and safe and had promising short- and mid-term results in almost all patients (angiographic success was not achieved in one patient with residual stenosis >20%, but similar to others, no intraprocedural complications occurred) (150). Similarly, in a case series of 13 patients who had stent underexpansion even with the use of appropriately sized dedicated very high-pressure/cutting balloons (≥30 atm), IVL successfully allowed MSA gain in all cases, with no in-hospital or 30-day MACE (151). IVL balloon sizing was guided by intravascular imaging.

In a recent retrospective study of 50 patients who underwent IVL, 13 patients had ISR as the target lesion. Angiographic success occurred in 100% and none experienced death, MACE, or major bleeding at 30 days (152). Most recently, a prospective multicenter observational study of IVL in calcified coronary lesions included ISR with stent underexpansion in almost 30% (*n* = 40) of the enrolled procedures. The primary endpoint of final procedural success was achieved in 97.5% of the ISR subgroup, with failure in only one patient (2.5%). Genuine MACE occurred in only two patients (one cardiac death due to procedure-related perforation, and another due to acute in-stent thrombosis), none of them in the ISR subgroup. Notably, rotablation was adjunctively used in 13.4% of the overall study population before IVL, and in 2.2% following IVL, suggesting that it could be complementary to IVL in particular situations to facilitate IVL balloon delivery or in case IVL was unable to adequately modify the lesion (153).

In cases where IVL is performed soon after the stenting procedure (within 6–12 months, if re-endothelialization is not yet complete), the impact on the integrity of the DES polymers is currently unknown and warrants evaluation (151, 154). Recently, Mousa and coworkers demonstrated in a case series of five patients with acute stent underexpansion in heavily calcified lesions, that angiographic success could be achieved in all cases, with no procedure-related MACE in the mid-term (follow-up ranged from 6 months to 24 months) (155). However, such theoretical risk of polymer damage might not be that relevant in the context of ISR, because by that time, most second-generation DES is fully re-endothelialized (156, 157).

In addition to peri-stent calcium with an underexpanded stent, calcified in-stent neoatherosclerosis can produce ISR (Waksman type II C). Reported cases show promising results with the use of IVL (complemented by rotational atherectomy in some instances) for managing such patients and illustrate the role of OCT in making the appropriate diagnosis and confirming an adequate result of IVL (158–160).

The other scenario is a well-expanded stent with excessive NIH (Waksman type II A), which is probably best treated with cutting or scoring balloons followed by DCB, an additional DES with an alternative drug (heterostenting), or vascular brachytherapy (VBT) (114).

Overall, DES and more recently DCB seem to be the most effective treatment approaches for ISR, backed by the results of several meta-analyses (161–164), with a recent collaborative meta-analysis pointing to a higher risk of TLR associated with paclitaxel-coated balloon (PCB) compared with DES, in the subgroup of patients with DES-ISR (165). It is worth noting that the enrolled trials in these meta-analyses included patients with both restenosed BMS and DES.

Tada et al. managed to show in a retrospective study that morphological assessment of the ISR tissue by OCT can guide the selection of the appropriate treatment strategy. This group compared different treatment strategies based on the OCT restenosis tissue characterization, in terms of mid-term ISR recurrence (re-ISR = binary restenosis on angiography) and TLR. The study population comprised a majority of patients with DES-ISR and a minority with BMS-ISR, and three strategies were compared: plain old balloon angioplasty (POBA) only, with high-pressure or scoring balloon inflated to almost rated burst pressure, POBA followed by PCB, or POBA followed by DES implantation and post-dilation. Importantly, PCB and DES performed equally across all tissue morphologies. Furthermore, in patients with homogeneous or high backscatter tissue pattern, PCI with POBA only was an independent predictor of re-ISR, which was attributed to the high proliferative activity of vascular smooth muscle cells in this lesion subset. Similarly, PCB and DES had lower ISR rates compared to POBA only, in patients with a layered neointima, but with no difference in TLR rates. Notably, such lesions include a certain amount of vascular smooth muscle cells in their adluminal layer. Contrarily, in patients with a heterogeneous or low backscatter neointima, there was no difference between the three treatment approaches, mostly due to the speculated excessive inflammation and hypersensitivity to drugs and/or polymers in this lesion pattern, where the use of a DES or PCB might aggravate inflammation (166). It is reasonable to infer that non-calcified neoatherosclerosis (Waksman type II B) can be approached in a similar manner (136).

Recently, the results of a pooled analysis of the OCT substudies of the RIBS IV and V prospective randomized clinical trials (patients with DES-ISR and BMS-ISR, respectively) have shown concurring results. The presence of neoatherosclerosis at the time of the index ISR procedure (detected by OCT) did not influence acute and long-term re-PCI outcomes (including angiographic and OCT outcomes at 6–9 months, and clinical outcomes at 3 years of follow-up). Moreover, these substudies suggested that both PCB and EES are effective and safe treatment options for ISR with neoatherosclerosis, although the limited sample size did not allow for more definitive conclusions as to the differential response to the chosen treatment (167).

When PCB was compared to EES, in the treatment of BMS ISR in 55 patients, OCT showed a better healing response in terms of stent strut coverage with PCB than EES, with no difference in the mean NIH area nor the luminal areas between the two treatments. Subsequently, there was no difference in the TLR rates either (168). Interestingly, Pleva et al. reported a significantly lower 12-month late luminal loss with PCB than with EES, when used to treat BMS restenosis, but with no difference in MACE (162). In contrast, the RIBS V trial has showed a lower rate of TLR at 3-year follow-up (with no difference in stent thrombosis rates) (169) with the use of EES than with PCB.

Bioresorbable vascular scaffolds (BVSs) emerged as another treatment option in ISR, where OCT proved to be helpful in optimizing implantation (170). However, late angiographic and clinical results were shown to be inferior to second generation DES (171–173).

In the very challenging subset of resistant/recurrent ISR, intracoronary imaging can be very helpful in delineating the number of stent layers at the lesion site, in addition to assessing the expansion of each stent layer. In the case of multiple layers of ISR, VBT is mostly the treatment of choice, as further stent layers are to be avoided (114, 174, 175). DCB is another option to avoid multiple metallic layers. However, it was shown recently to be less effective for ISR lesions with three or more previously implanted stent layers, with significantly higher rates of the primary endpoint of MACE (mainly driven by TLR) and the secondary endpoint of TLR, both >40% at 1 year (176). Waksman et al. in their algorithm adopt the same approach that in the presence of >2 layers of stent (Waksman type V), an additional layer of stent should better be avoided (136). In cases where DES implantation is used to treat ISR, it has been illustrated by IVUS that stent underexpansion is a significant cause of recurrence, which highlights the importance of intravascular imaging to ensure adequate stent expansion (177).

In keeping with this, Yin et al. explored in a retrospective observational study the ISR characteristics by OCT that lead to repeat DES stenting underexpansion. The study included restenotic BMS, as well as first- and second-generation DES. Old stent underexpansion (MSA <4.5 mm^2^ and expansion <70%), multiple layers of old stent, significant neointimal, or peri-stent calcium (maximum calcium angle >180° and/or maximum calcium thickness >0.5 mm) were all independently associated with new stent underexpansion, which in turn had a higher rate of myocardial infarction and TLR at 2 years (178). Therefore, in cases with significant calcium, disruption by ELCA or IVL should be considered to facilitate full expansion of the new stent, akin to *de novo* stenting.

Another subset for recurrent ISR is stent fracture (Waksman type I B), where repeat stenting with an EES or ZES was non-different from the use of DCB, in terms of 12-month binary re-restenosis and repeat TLR, which were unfortunately high in both groups (179). In contrast, another prospective large-scale study that assigned treatment for stent fracture-related ISR, depending on the fracture angiographic subtype (180) (repeat DES for type I/II, and balloon angioplasty for type III/IV), had lower repeat ISR and repeat TLR rates, which were not different from those in the non-fracture comparator group (102). In either study, intravascular imaging did not inform the choice of treatment, and therefore, its added value in that respect remains unexplored.

## Knowledge gaps

Definitions of ISR based on intracoronary imaging need to be further validated in terms of clinical outcomes, to replace the angiographic restenosis that is as yet too often used as a study endpoint.

The most effective protocol (and tool) for imaging-guided stent implantation has to be determined, to avert the key mechanical problem of stent underexpansion but also to best recognize other predictors of stent failure. For instance, different intravascular imaging-based stent sizing approaches were proposed, including more conservative vs. more aggressive approaches, for selection of the appropriate stent diameter, but they have not been compared head-to-head, in terms of feasibility and clinical outcomes including ISR and clinically driven TLR.

Once the ISR has set in, there is a lack of evidence as to whether an individualized/tailored ISR management approach based on the underlying substrate/mechanism detected by intravascular imaging has an impact on clinical outcomes. In other words, treatment recommendations are largely based on the observational data and expert consensus rather than randomized controlled trials. Therefore, the added value of the systematic use of intracoronary imaging in guiding re-interventions is still unsettled. The results of the ongoing ILUMIEN IV trial (NCT03507777) and IMPROVE trial (NCT04221815) will help to better define the role of OCT and IVUS, respectively, in this regard (69, 181). An ideal treatment-oriented imaging-based classification would in particular enable the identification of lesions in which DCB angioplasty can provide sufficiently good outcomes without the need for additional stent implantation.

Newer technologies such as high-definition ultrasound warrant further head-to-head comparisons with established technologies such as OCT, in terms of PCI guidance, and impact on clinical outcomes.

## Future directions

The cost-effectiveness of IVUS, particularly in patients at a greater risk of restenosis, has been shown (182). However, similar studies are needed for OCT to overcome the financial barrier in countries where the additional cost is prohibitive because of inadequate reimbursement. This will promote wider adoption and integration into routine practice.

Refinements in technology, such as lower-profile and more deliverable imaging catheters, faster pullbacks, higher resolution, and fully automated software to support online assessment, are expected to make the imaging technology more user-friendly and more widely adopted.

A recently developed 60-MHz high-definition IVUS (HD-IVUS) system tends to combine the advantages of IVUS and OCT, in the sense that it offers superior axial resolution compared to contemporary 40–45 MHz IVUS transducers (<40 vs. ∼100 μm), faster catheter pullback speeds (10 vs. 0.5 mm/s), and more temporal resolution (60 vs. 30 frames/second) and yet maintains the advantage of greater imaging penetration relative to OCT, with visualization of the vessel wall (EEL) (183). Although data are still preliminary and derived from small-sized studies, each of HD-IVUS and OCT seems to maintain some of its original strengths. For example, in a prospective study of 29 lesions in 29 patients, HD-IVUS had an excellent concordance with OCT on luminal area measurements, both pre- and post-intervention. However, before intervention, HD-IVUS offered better visualization of the EEL. Moreover, after intervention, OCT more frequently identified TP, stent-edge dissection, and ASM. It is still unclear whether this additional morphological information provided by OCT compared to HD-IVUS has clinical implications (184).

Another interesting development is the ultrafast single cardiac cycle OCT system developed by Kim et al. with a pullback speed of 100 mm/s, with the prospective ECG triggering technology, which enabled imaging of long coronary segments of a swine *in vivo* within one cardiac cycle, with minimal cardiac motion artifact. This technology has yet to be translated to clinical use (185).

In an attempt to obtain more physiologic insight by IVUS after stenting, it has been retrospectively shown that the difference in intraluminal intensity of blood speckle on IB-IVUS between the ostium of the target vessel and the distal reference of the implanted stent may predict TVR, based on its relationship to post-procedural FFR (186). This approach needs to be prospectively tested.

## Author contributions

AA wrote the first draft of the manuscript. TG provided figures and supervision. Both authors are responsible for further revisions. Both authors contributed to the article and approved the submitted version.

## References

[1] Dangas GD Claessen BE Caixeta A Sanidas EA Mintz GS Mehran R. In-stent restenosis in the drug-eluting stent era. *J Am Coll Cardiol.* (2010) 56:1897–907. 21109112 10.1016/j.jacc.2010.07.028

[2] Park DW Hong MK Mintz GS Lee CW Song JM Han KH Two-year follow-up of the quantitative angiographic and volumetric intravascular ultrasound analysis after nonpolymeric paclitaxel-eluting stent implantation: Late “catch-up” phenomenon from ASPECT Study. *J Am Coll Cardiol.* (2006) 48:2432–9. 10.1016/j.jacc.2006.08.033 17174179

[3] Buccheri D Piraino D Andolina G Cortese B. Understanding and managing in-stent restenosis: A review of clinical data, from pathogenesis to treatment. *J Thorac Dis.* (2016) 8:E1150–62. 27867580 10.21037/jtd.2016.10.93 PMC5107494

[4] Perera D Postema P Rashid R Patel S Blows L Marber M Does a well developed collateral circulation predispose to restenosis after percutaneous coronary intervention? An intravascular ultrasound study. *Heart.* (2006) 92:763–7. 10.1136/hrt.2005.067322 16216859 PMC1860667

[5] Roubin GS King SB III Douglas JS Jr. Restenosis after percutaneous transluminal coronary angioplasty: The Emory University Hospital experience. *Am J Cardiol.* (1987) 60:39B–43B. 10.1016/0002-9149(87)90482-6 2956840

[6] Gould KL Lipscomb K Hamilton GW. Physiologic basis for assessing critical coronary stenosis. Instantaneous flow response and regional distribution during coronary hyperemia as measures of coronary flow reserve. *Am J Cardiol.* (1974) 33:87–94. 10.1016/0002-9149(74)90743-7 4808557

[7] Cutlip DE Chauhan MS Baim DS Ho KK Popma JJ Carrozza JP Clinical restenosis after coronary stenting: Perspectives from multicenter clinical trials. *J Am Coll Cardiol.* (2002) 40:2082–9. 12505217 10.1016/s0735-1097(02)02597-4

[8] Rosenschein U Topol EJ. Uncoupling clinical outcomes and coronary angiography: A review and perspective of recent trials in coronary artery disease. *Am Heart J.* (1996) 132:910–20. 10.1016/s0002-8703(96)90335-x 8831390

[9] Kuntz RE Baim DS. Defining coronary restenosis. Newer clinical and angiographic paradigms. *Circulation.* (1993) 88:1310–23. 10.1161/01.cir.88.3.1310 8353892

[10] Mauri L Orav EJ Kuntz RE. Late loss in lumen diameter and binary restenosis for drug-eluting stent comparison. *Circulation.* (2005) 111:3435–42. 15967844 10.1161/CIRCULATIONAHA.104.513952

[11] Garcia-Garcia HM Shen Z Piazza N. Study of restenosis in drug eluting stents: New insights from greyscale intravascular ultrasound and virtual histology. *EuroIntervention.* (2009) 5:D84–92. 19736078

[12] Byrne RA Joner M Alfonso F Kastrati A. Treatment of in-stent restenosis. In: Bhatt DL editor. *Cardiovascular intervention: A companion to Braunwald’s heart disease.* (Amsterdam: Elsevier) (2016). p. 209–22.

[13] Nishioka T Amanullah AM Luo H Berglund H Kim CJ Nagai T Clinical validation of intravascular ultrasound imaging for assessment of coronary stenosis severity: Comparison with stress myocardial perfusion imaging. *J Am Coll Cardiol.* (1999) 33:1870–8. 10362187 10.1016/s0735-1097(99)00100-x

[14] Abizaid A Mintz GS Pichard AD Kent KM Satler LF Walsh CL Clinical, intravascular ultrasound, and quantitative angiographic determinants of the coronary flow reserve before and after percutaneous transluminal coronary angioplasty. *Am J Cardiol.* (1998) 82:423–8. 9723627 10.1016/s0002-9149(98)00355-5

[15] Nogic J Prosser H O’Brien J Thakur U Soon K Proimos G The assessment of intermediate coronary lesions using intracoronary imaging. *Cardiovasc Diagn Ther.* (2020) 10:1445–60. 33224767 10.21037/cdt-20-226 PMC7666953

[16] Cutlip DE Windecker S Mehran R Boam A Cohen DJ van Es GA Clinical end points in coronary stent trials: A case for standardized definitions. *Circulation.* (2007) 115:2344–51. 10.1161/CIRCULATIONAHA.106.685313 17470709

[17] Garcia-Garcia HM McFadden EP Farb A Mehran R Stone GW Spertus J Standardized end point definitions for coronary intervention trials: The academic research consortium-2 consensus document. *Circulation.* (2018) 137:2635–50. 29891620 10.1161/CIRCULATIONAHA.117.029289

[18] Oemrawsingh PV Mintz GS Schalij MJ Zwinderman AH Jukema JW van der Wall EE Intravascular ultrasound guidance improves angiographic and clinical outcome of stent implantation for long coronary artery stenoses: Final results of a randomized comparison with angiographic guidance (TULIP Study). *Circulation.* (2003) 107:62–7. 10.1161/01.cir.0000043240.87526.3f 12515744

[19] Fitzgerald PJ Oshima A Hayase M Metz JA Bailey SR Baim DS Final results of the can routine ultrasound influence stent expansion (CRUISE) study. *Circulation.* (2000) 102:523–30. 10.1161/01.cir.102.5.523 10920064

[20] Parise H Maehara A Stone GW Leon MB Mintz GS. Meta-analysis of randomized studies comparing intravascular ultrasound versus angiographic guidance of percutaneous coronary intervention in pre-drug-eluting stent era. *Am J Cardiol.* (2011) 107:374–82. 10.1016/j.amjcard.2010.09.030 21257001

[21] Hur SH Kang SJ Kim YH Ahn JM Park DW Lee SW Impact of intravascular ultrasound-guided percutaneous coronary intervention on long-term clinical outcomes in a real world population. *Catheter Cardiovasc Interv.* (2013) 81:407–16. 10.1002/ccd.23279 21805605

[22] Zhang Y Farooq V Garcia-Garcia HM Bourantas CV Tian N Dong S Comparison of intravascular ultrasound versus angiography-guided drug-eluting stent implantation: A meta-analysis of one randomised trial and ten observational studies involving 19,619 patients. *EuroIntervention.* (2012) 8:855–65. 23171805 10.4244/EIJV8I7A129

[23] Ahn JM Kang SJ Yoon SH Park HW Kang SM Lee JY Meta-analysis of outcomes after intravascular ultrasound-guided versus angiography-guided drug-eluting stent implantation in 26,503 patients enrolled in three randomized trials and 14 observational studies. *Am J Cardiol.* (2014) 113:1338–47. 10.1016/j.amjcard.2013.12.043 24685326

[24] Jang JS Song YJ Kang W Jin HY Seo JS Yang TH Intravascular ultrasound-guided implantation of drug-eluting stents to improve outcome: A meta-analysis. *JACC Cardiovasc Interv.* (2014) 7:233–43. 24529934 10.1016/j.jcin.2013.09.013

[25] Maehara A Mintz GS Witzenbichler B Weisz G Neumann FJ Rinaldi MJ Relationship between intravascular ultrasound guidance and clinical outcomes after drug-eluting stents. *Circ Cardiovasc Interv.* (2018) 11:e006243. 10.1161/CIRCINTERVENTIONS.117.006243 30571206

[26] Kang SJ Mintz GS Akasaka T Park DW Lee JY Kim WJ Optical coherence tomographic analysis of in-stent neoatherosclerosis after drug-eluting stent implantation. *Circulation.* (2011) 123:2954–63. 21646494 10.1161/CIRCULATIONAHA.110.988436

[27] Buccheri S Franchina G Romano S Puglisi S Venuti G D’Arrigo P Clinical outcomes following intravascular imaging-guided versus coronary angiography-guided percutaneous coronary intervention with stent implantation: A systematic review and bayesian network meta-analysis of 31 studies and 17,882 patients. *JACC Cardiovasc Interv.* (2017) 10:2488–98. 10.1016/j.jcin.2017.08.051 29153502

[28] Chieffo A Latib A Caussin C Presbitero P Galli S Menozzi A A prospective, randomized trial of intravascular-ultrasound guided compared to angiography guided stent implantation in complex coronary lesions: The AVIO trial. *Am Heart J.* (2013) 165:65–72. 10.1016/j.ahj.2012.09.017 23237135

[29] Choi KH Song YB Lee JM Lee SY Park TK Yang JH Impact of intravascular ultrasound-guided percutaneous coronary intervention on long-term clinical outcomes in patients undergoing complex procedures. *JACC Cardiovasc Interv.* (2019) 12:607–20. 30878474 10.1016/j.jcin.2019.01.227

[30] Witzenbichler B Maehara A Weisz G Neumann FJ Rinaldi MJ Metzger DC Relationship between intravascular ultrasound guidance and clinical outcomes after drug-eluting stents: The assessment of dual antiplatelet therapy with drug-eluting stents (ADAPT-DES) study. *Circulation.* (2014) 129:463–70. 24281330 10.1161/CIRCULATIONAHA.113.003942

[31] Hong SJ Kim BK Shin DH Nam CM Kim JS Ko YG Effect of intravascular ultrasound-guided vs angiography-guided everolimus-eluting stent implantation: The IVUS-XPL randomized clinical trial. *JAMA.* (2015) 314:2155–63. 26556051 10.1001/jama.2015.15454

[32] Zhang J Gao X Kan J Ge Z Han L Lu S Intravascular ultrasound versus angiography-guided drug-eluting stent implantation: The ULTIMATE trial. *J Am Coll Cardiol.* (2018) 72:3126–37. 30261237 10.1016/j.jacc.2018.09.013

[33] Hong SJ Mintz GS Ahn CM Kim JS Kim BK Ko YG Effect of intravascular ultrasound-guided drug-eluting stent implantation: 5-year follow-up of the IVUS-XPL randomized trial. *JACC Cardiovasc Interv.* (2020) 13:62–71. 10.1016/j.jcin.2019.09.033 31918944

[34] Gao XF Ge Z Kong XQ Kan J Han L Lu S 3-year outcomes of the ultimate Trial comparing intravascular ultrasound versus angiography-guided drug-eluting stent implantation. *JACC Cardiovasc Interv.* (2021) 14:247–57. 33541535 10.1016/j.jcin.2020.10.001

[35] Prati F Di Vito L Biondi-Zoccai G Occhipinti M La Manna A Tamburino C Angiography alone versus angiography plus optical coherence tomography to guide decision-making during percutaneous coronary intervention: The Centro per la lotta contro l’infarto-optimisation of percutaneous coronary intervention (CLI-OPCI) study. *EuroIntervention.* (2012) 8:823–9. 10.4244/EIJV8I7A125 23034247

[36] Prati F Romagnoli E Burzotta F Limbruno U Gatto L La Manna A Clinical impact of OCT findings during PCI: The CLI-OPCI II study. *JACC Cardiovasc Imaging.* (2015) 8:1297–305. 10.1016/j.jcmg.2015.08.013 26563859

[37] Jones DA Rathod KS Koganti S Hamshere S Astroulakis Z Lim P Angiography alone versus angiography plus optical coherence tomography to guide percutaneous coronary intervention: Outcomes from the Pan-London PCI Cohort. *JACC Cardiovasc Interv.* (2018) 11:1313–21. 10.1016/j.jcin.2018.01.274 30025725

[38] Meneveau N Souteyrand G Motreff P Caussin C Amabile N Ohlmann P Optical coherence tomography to optimize results of percutaneous coronary intervention in patients with non-st-elevation acute coronary syndrome: Results of the multicenter, randomized doctors study (does optical coherence tomography optimize results of stenting). *Circulation.* (2016) 134:906–17. 27573032 10.1161/CIRCULATIONAHA.116.024393

[39] Kubo T Shinke T Okamura T Hibi K Nakazawa G Morino Y Optical frequency domain imaging vs. intravascular ultrasound in percutaneous coronary intervention (OPINION trial): One-year angiographic and clinical results. *Eur Heart J.* (2017) 38:3139–47. 10.1093/eurheartj/ehx351 29121226 PMC5837511

[40] Mintz GS. Features and parameters of drug-eluting stent deployment discoverable by intravascular ultrasound. *Am J Cardiol.* (2007) 100:26M–35M. 10.1016/j.amjcard.2007.08.019 17950829

[41] Moussa I Moses J Di Mario C Albiero R De Gregorio J Adamian M Does the specific intravascular ultrasound criterion used to optimize stent expansion have an impact on the probability of stent restenosis? *Am J Cardiol.* (1999) 83:1012–7. 10.1016/s0002-9149(99)00006-5 10190511

[42] Doi H Maehara A Mintz GS Yu A Wang H Mandinov L Impact of post-intervention minimal stent area on 9-month follow-up patency of paclitaxel-eluting stents: An integrated intravascular ultrasound analysis from the TAXUS IV, V, and VI and TAXUS ATLAS workhorse, long lesion, and direct stent trials. *JACC Cardiovasc Interv.* (2009) 2:1269–75. 10.1016/j.jcin.2009.10.005 20129555

[43] Hong MK Mintz GS Lee CW Park DW Choi BR Park KH Intravascular ultrasound predictors of angiographic restenosis after sirolimus-eluting stent implantation. *Eur Heart J.* (2006) 27:1305–10. 16682378 10.1093/eurheartj/ehi882

[44] Song HG Kang SJ Ahn JM Kim WJ Lee JY Park DW Intravascular ultrasound assessment of optimal stent area to prevent in-stent restenosis after zotarolimus-, everolimus-, and sirolimus-eluting stent implantation. *Catheter Cardiovasc Interv.* (2014) 83:873–8. 10.1002/ccd.24560 22815193

[45] Fujimura T Matsumura M Witzenbichler B Metzger DC Rinaldi MJ Duffy PL Stent expansion indexes to predict clinical outcomes: An IVUS substudy from ADAPT-DES. *JACC Cardiovasc Interv.* (2021) 14:1639–50. 10.1016/j.jcin.2021.05.019 34353595

[46] Kang SJ Ahn JM Song H Kim WJ Lee JY Park DW Comprehensive intravascular ultrasound assessment of stent area and its impact on restenosis and adverse cardiac events in 403 patients with unprotected left main disease. *Circ Cardiovasc Interv.* (2011) 4:562–9. 10.1161/CIRCINTERVENTIONS.111.964643 22045969

[47] Werner GS Gastmann O Ferrari M Scholz KH Schunemann S Figulla HR. Determinants of stent restenosis in chronic coronary occlusions assessed by intracoronary ultrasound. *Am J Cardiol.* (1999) 83:1164–9. 10215277 10.1016/s0002-9149(99)00052-1

[48] Choi SY Maehara A Cristea E Witzenbichler B Guagliumi G Brodie B Usefulness of minimum stent cross sectional area as a predictor of angiographic restenosis after primary percutaneous coronary intervention in acute myocardial infarction (from the HORIZONS-AMI Trial IVUS substudy). *Am J Cardiol.* (2012) 109:455–60. 10.1016/j.amjcard.2011.10.005 22118823

[49] Soeda T Uemura S Park SJ Jang Y Lee S Cho JM Incidence and Clinical significance of poststent optical coherence tomography findings: One-year follow-up study from a multicenter registry. *Circulation.* (2015) 132:1020–9. 10.1161/CIRCULATIONAHA.114.014704 26162917

[50] Maehara A Matsumura M Ali ZA Mintz GS Stone GWIVUS-. Guided versus OCT-guided coronary stent implantation: A critical appraisal. *JACC Cardiovasc Imaging.* (2017) 10:1487–503. 10.1016/j.jcmg.2017.09.008 29216976

[51] Kubo T Akasaka T Shite J Suzuki T Uemura S Yu B OCT compared with IVUS in a coronary lesion assessment: The OPUS-CLASS study. *JACC Cardiovasc Imaging.* (2013) 6:1095–104. 10.1016/j.jcmg.2013.04.014 24011777

[52] Habara M Nasu K Terashima M Kaneda H Yokota D Ko E Impact of frequency-domain optical coherence tomography guidance for optimal coronary stent implantation in comparison with intravascular ultrasound guidance. *Circ Cardiovasc Interv.* (2012) 5:193–201. 22456026 10.1161/CIRCINTERVENTIONS.111.965111

[53] Maehara A Ben-Yehuda O Ali Z Wijns W Bezerra HG Shite J Comparison of stent expansion guided by optical coherence tomography versus intravascular ultrasound: The ILUMIEN II study (observational study of optical coherence tomography [OCT] in patients undergoing fractional flow reserve [FFR] and percutaneous coronary intervention). *JACC Cardiovasc Interv.* (2015) 8:1704–14. 10.1016/j.jcin.2015.07.024 26585621

[54] Ali ZA Maehara A Genereux P Shlofmitz RA Fabbiocchi F Nazif TM Optical coherence tomography compared with intravascular ultrasound and with angiography to guide coronary stent implantation (ILUMIEN III: OPTIMIZE PCI): A randomised controlled trial. *Lancet.* (2016) 388:2618–28. 27806900 10.1016/S0140-6736(16)31922-5

[55] Mintz GS Popma JJ Pichard AD Kent KM Satler LF Chuang YC Patterns of calcification in coronary artery disease. A statistical analysis of intravascular ultrasound and coronary angiography in 1155 lesions. *Circulation.* (1995) 91:1959–65. 10.1161/01.cir.91.7.1959 7895353

[56] Kostamaa H Donovan J Kasaoka S Tobis J Fitzpatrick L. Calcified plaque cross-sectional area in human arteries: Correlation between intravascular ultrasound and undecalcified histology. *Am Heart J.* (1999) 137:482–8. 10.1016/s0002-8703(99)70496-5 10047630

[57] Friedrich GJ Moes NY Muhlberger VA Gabl C Mikuz G Hausmann D Detection of intralesional calcium by intracoronary ultrasound depends on the histologic pattern. *Am Heart J.* (1994) 128:435–41. 10.1016/0002-8703(94)90614-9 8074002

[58] Kobayashi Y Okura H Kume T Yamada R Kobayashi Y Fukuhara K Impact of target lesion coronary calcification on stent expansion. *Circ J.* (2014) 78:2209–14. 25017740 10.1253/circj.cj-14-0108

[59] Maejima N Hibi K Saka K Akiyama E Konishi M Endo M Relationship between thickness of calcium on optical coherence tomography and crack formation after balloon dilatation in calcified plaque requiring rotational atherectomy. *Circ J.* (2016) 80:1413–9. 10.1253/circj.CJ-15-1059 27087360

[60] Fujino A Mintz GS Matsumura M Lee T Kim SY Hoshino M A new optical coherence tomography-based calcium scoring system to predict stent underexpansion. *EuroIntervention.* (2018) 13:e2182–9. 10.4244/EIJ-D-17-00962 29400655

[61] Kubo T Shimamura K Ino Y Yamaguchi T Matsuo Y Shiono Y Superficial calcium fracture after PCI as assessed by OCT. *JACC Cardiovasc Imaging.* (2015) 8:1228–9. 10.1016/j.jcmg.2014.11.012 25797130

[62] Park H Ahn JM Kang DY Lee JB Park S Ko E Optimal stenting technique for complex coronary lesions: Intracoronary imaging-guided pre-dilation, stent sizing, and post-dilation. *JACC Cardiovasc Interv.* (2020) 13:1403–13. 10.1016/j.jcin.2020.03.023 32473888

[63] Kim BK Shin DH Hong MK Park HS Rha SW Mintz GS Clinical impact of intravascular ultrasound-guided chronic total occlusion intervention with zotarolimus-eluting versus biolimus-eluting stent implantation: Randomized study. *Circ Cardiovasc Interv.* (2015) 8:e002592. 10.1161/CIRCINTERVENTIONS.115.002592 26156151

[64] Shlofmitz E Torguson R Zhang C Craig PE Mintz GS Khalid N Impact of intravascular ultrasound on outcomes following percutaneous coronary intervention in complex lesions (iOPEN complex). *Am Heart J.* (2020) 221:74–83. 10.1016/j.ahj.2019.12.008 31951847

[65] Tian NL Gami SK Ye F Zhang JJ Liu ZZ Lin S Angiographic and clinical comparisons of intravascular ultrasound- versus angiography-guided drug-eluting stent implantation for patients with chronic total occlusion lesions: Two-year results from a randomised AIR-CTO study. *EuroIntervention.* (2015) 10:1409–17. 10.4244/EIJV10I12A245 25912391

[66] Hong SJ Kim BK Shin DH Kim JS Hong MK Gwon HC Usefulness of intravascular ultrasound guidance in percutaneous coronary intervention with second-generation drug-eluting stents for chronic total occlusions (from the multicenter Korean-chronic total occlusion registry). *Am J Cardiol.* (2014) 114:534–40. 10.1016/j.amjcard.2014.05.027 25001153

[67] Raber L Mintz GS Koskinas KC Johnson TW Holm NR Onuma Y Clinical use of intracoronary imaging. Part 1: Guidance and optimization of coronary interventions. An expert consensus document of the European association of percutaneous cardiovascular interventions. *Eur Heart J.* (2018) 39:3281–300. 29790954 10.1093/eurheartj/ehy285

[68] Ali ZA Karimi Galougahi K Maehara A Shlofmitz RA Fabbiocchi F Guagliumi G Outcomes of optical coherence tomography compared with intravascular ultrasound and with angiography to guide coronary stent implantation: One-year results from the ILUMIEN III: OPTIMIZE PCI trial. *EuroIntervention.* (2021) 16:1085–91. 10.4244/EIJ-D-20-00498 32540793 PMC9724851

[69] Ali Z Landmesser U Karimi Galougahi K Maehara A Matsumura M Shlofmitz RA Optical coherence tomography-guided coronary stent implantation compared to angiography: A multicentre randomised trial in PCI – design and rationale of ILUMIEN IV: OPTIMAL PCI. *EuroIntervention.* (2021) 16:1092–9. 10.4244/EIJ-D-20-00501 32863246 PMC9725042

[70] Costa MA Angiolillo DJ Tannenbaum M Driesman M Chu A Patterson J Impact of stent deployment procedural factors on long-term effectiveness and safety of sirolimus-eluting stents (final results of the multicenter prospective STLLR trial). *Am J Cardiol.* (2008) 101:1704–11. 10.1016/j.amjcard.2008.02.053 18549844

[71] Sakurai R Ako J Morino Y Sonoda S Kaneda H Terashima M Predictors of edge stenosis following sirolimus-eluting stent deployment (a quantitative intravascular ultrasound analysis from the SIRIUS trial). *Am J Cardiol.* (2005) 96:1251–3. 10.1016/j.amjcard.2005.06.066 16253592

[72] Liu J Maehara A Mintz GS Weissman NJ Yu A Wang H An integrated TAXUS IV, V, and VI intravascular ultrasound analysis of the predictors of edge restenosis after bare metal or paclitaxel-eluting stents. *Am J Cardiol.* (2009) 103:501–6. 19195510 10.1016/j.amjcard.2008.10.010

[73] Kang SJ Cho YR Park GM Ahn JM Kim WJ Lee JY Intravascular ultrasound predictors for edge restenosis after newer generation drug-eluting stent implantation. *Am J Cardiol.* (2013) 111:1408–14. 10.1016/j.amjcard.2013.01.288 23433757

[74] Ino Y Kubo T Matsuo Y Yamaguchi T Shiono Y Shimamura K Optical coherence tomography predictors for edge restenosis after everolimus-eluting stent implantation. *Circ Cardiovasc Interv.* (2016) 9:e004231. 10.1161/CIRCINTERVENTIONS.116.004231 27688261

[75] Koyama K. A prospective, single-center, ramdomized study to assess whether co-registration of OCT and angiography can reduce geographic miss. (2016). TCTMD; October. 10.1002/ccd.27854 30345635

[76] Schwartz RS Huber KC Murphy JG Edwards WD Camrud AR Vlietstra RE Restenosis and the proportional neointimal response to coronary artery injury: Results in a porcine model. *J Am Coll Cardiol.* (1992) 19:267–74. 10.1016/0735-1097(92)90476-4 1732351

[77] Kobayashi N Mintz GS Witzenbichler B Metzger DC Rinaldi MJ Duffy PL Prevalence, features, and prognostic importance of edge dissection after drug-eluting stent implantation: An ADAPT-DES intravascular ultrasound substudy. *Circ Cardiovasc Interv.* (2016) 9:e003553. 10.1161/CIRCINTERVENTIONS.115.003553 27402854

[78] Radu MD Raber L Heo J Gogas BD Jorgensen E Kelbaek H Natural history of optical coherence tomography-detected non-flow-limiting edge dissections following drug-eluting stent implantation. *EuroIntervention.* (2014) 9:1085–94. 10.4244/EIJV9I9A183 24064426

[79] Hong MK Park SW Lee NH Nah DY Lee CW Kang DH Long-term outcomes of minor dissection at the edge of stents detected with intravascular ultrasound. *Am J Cardiol.* (2000) 86:791–5,A9. 10.1016/s0002-9149(00)01085-7 11018205

[80] Sheris SJ Canos MR Weissman NJ. Natural history of intravascular ultrasound-detected edge dissections from coronary stent deployment. *Am Heart J.* (2000) 139:59–63. 10.1016/s0002-8703(00)90309-0 10618563

[81] Kume T Okura H Miyamoto Y Yamada R Saito K Tamada T Natural history of stent edge dissection, tissue protrusion and incomplete stent apposition detectable only on optical coherence tomography after stent implantation - preliminary observation. *Circ J.* (2012) 76:698–703. 10.1253/circj.cj-11-0845 22251751

[82] Chamie D Bezerra HG Attizzani GF Yamamoto H Kanaya T Stefano GT Incidence, predictors, morphological characteristics, and clinical outcomes of stent edge dissections detected by optical coherence tomography. *JACC Cardiovasc Interv.* (2013) 6:800–13. 10.1016/j.jcin.2013.03.019 23871510

[83] De Cock D Bennett J Ughi GJ Dubois C Sinnaeve P Dhooge J Healing course of acute vessel wall injury after drug-eluting stent implantation assessed by optical coherence tomography. *Eur Heart J Cardiovasc Imaging.* (2014) 15:800–9. 24497520 10.1093/ehjci/jeu003

[84] Kawamori H Shite J Shinke T Otake H Matsumoto D Nakagawa M Natural consequence of post-intervention stent malapposition, thrombus, tissue prolapse, and dissection assessed by optical coherence tomography at mid-term follow-up. *Eur Heart J Cardiovasc Imaging.* (2013) 14:865–75. 10.1093/ehjci/jes299 23291393 PMC3738096

[85] Prati F Romagnoli E La Manna A Burzotta F Gatto L Marco V Long-term consequences of optical coherence tomography findings during percutaneous coronary intervention: The centro per la lotta contro l’infarto – Optimization of percutaneous coronary intervention (CLI-OPCI) LATE study. *EuroIntervention.* (2018) 14:e443–51. 10.4244/EIJ-D-17-01111 29633940

[86] Bouki KP Sakkali E Toutouzas K Vlad D Barmperis D Phychari S Impact of coronary artery stent edge dissections on long-term clinical outcome in patients with acute coronary syndrome: An optical coherence tomography study. *Catheter Cardiovasc Interv.* (2015) 86:237–46. 10.1002/ccd.25855 25620191

[87] van Zandvoort LJC Tomaniak M Tovar Forero MN Masdjedi K Visseren L Witberg K Predictors for clinical outcome of untreated stent edge dissections as detected by optical coherence tomography. *Circ Cardiovasc Interv.* (2020) 13:e008685. 10.1161/CIRCINTERVENTIONS.119.008685 32089001

[88] Hong MK Park SW Lee CW Kang DH Song JK Kim JJ Long-term outcomes of minor plaque prolapsed within stents documented with intravascular ultrasound. *Catheter Cardiovasc Interv.* (2000) 51:22–6. 10.1002/1522-726x(200009)51:1<22::aid-ccd6>3.0.co;2-i 10973013

[89] Futamatsu H Sabate M Angiolillo DJ Jimenez-Quevedo P Corros C Morikawa-Futamatsu K Characterization of plaque prolapse after drug-eluting stent implantation in diabetic patients: A three-dimensional volumetric intravascular ultrasound outcome study. *J Am Coll Cardiol.* (2006) 48:1139–45. 10.1016/j.jacc.2006.05.050 16978996

[90] Qiu F Mintz GS Witzenbichler B Metzger DC Rinaldi MJ Duffy PL Prevalence and clinical impact of tissue protrusion after stent implantation: An ADAPT-DES intravascular ultrasound substudy. *JACC Cardiovasc Interv.* (2016) 9:1499–507. 10.1016/j.jcin.2016.05.043 27478119

[91] Gutierrez-Chico JL Wykrzykowska J Nuesch E van Geuns RJ Koch KT Koolen JJ Vascular tissue reaction to acute malapposition in human coronary arteries: Sequential assessment with optical coherence tomography. *Circ Cardiovasc Interv.* (2012) 5:S1–8. 10.1161/CIRCINTERVENTIONS.111.965301 22319063

[92] Shimamura K Kubo T Akasaka T Kozuma K Kimura K Kawamura M Outcomes of everolimus-eluting stent incomplete stent apposition: A serial optical coherence tomography analysis. *Eur Heart J Cardiovasc Imaging.* (2015) 16:23–8. 25342855 10.1093/ehjci/jeu174

[93] Sotomi Y Onuma Y Dijkstra J Miyazaki Y Kozuma K Tanabe K Fate of post-procedural malapposition of everolimus-eluting polymeric bioresorbable scaffold and everolimus-eluting cobalt chromium metallic stent in human coronary arteries: Sequential assessment with optical coherence tomography in ABSORB Japan trial. *Eur Heart J Cardiovasc Imaging.* (2018) 19:59–66. 10.1093/ehjci/jew329 28158421

[94] Im E Kim BK Ko YG Shin DH Kim JS Choi D Incidences, predictors, and clinical outcomes of acute and late stent malapposition detected by optical coherence tomography after drug-eluting stent implantation. *Circ Cardiovasc Interv.* (2014) 7:88–96. 10.1161/CIRCINTERVENTIONS.113.000797 24425586

[95] Romagnoli E Gatto L La Manna A Burzotta F Taglieri N Saia F Role of residual acute stent malapposition in percutaneous coronary interventions. *Catheter Cardiovasc Interv.* (2017) 90:566–75. 28295990 10.1002/ccd.26974

[96] Prati F Romagnoli E Gatto L La Manna A Burzotta F Limbruno U Clinical impact of suboptimal stenting and residual intrastent plaque/thrombus protrusion in patients with acute coronary syndrome: The CLI-OPCI ACS substudy (centro per la lotta contro l’infarto-optimization of percutaneous coronary intervention in acute coronary syndrome). *Circ Cardiovasc Interv.* (2016) 9:e003726. 10.1161/CIRCINTERVENTIONS.115.003726 27965297

[97] Steinberg DH Mintz GS Mandinov L Yu A Ellis SG Grube E Long-term impact of routinely detected early and late incomplete stent apposition: An integrated intravascular ultrasound analysis of the TAXUS IV, V, and VI and TAXUS ATLAS workhorse, long lesion, and direct stent studies. *JACC Cardiovasc Interv.* (2010) 3:486–94. 10.1016/j.jcin.2010.03.007 20488404

[98] Wang B Mintz GS Witzenbichler B Souza CF Metzger DC Rinaldi MJ Predictors and long-term clinical impact of acute stent malapposition: An assessment of dual antiplatelet therapy with drug-eluting stents (ADAPT-DES) intravascular ultrasound substudy. *J Am Heart Assoc.* (2016) 5:e004438. 10.1161/JAHA.116.004438 28007741 PMC5210413

[99] Inaba S Mintz GS Yun KH Yakushiji T Shimizu T Kang SJ Mechanical complications of everolimus-eluting stents associated with adverse events: An intravascular ultrasound study. *EuroIntervention.* (2014) 9:1301–8. 10.4244/EIJV9I11A220 24650772

[100] Chakravarty T White AJ Buch M Naik H Doctor N Schapira J Meta-analysis of incidence, clinical characteristics and implications of stent fracture. *Am J Cardiol.* (2010) 106:1075–80. 10.1016/j.amjcard.2010.06.010 20920641

[101] Kuramitsu S Iwabuchi M Haraguchi T Domei T Nagae A Hyodo M Incidence and clinical impact of stent fracture after everolimus-eluting stent implantation. *Circ Cardiovasc Interv.* (2012) 5:663–71. 23011266 10.1161/CIRCINTERVENTIONS.112.969238

[102] Kan J Ge Z Zhang JJ Liu ZZ Tian NL Ye F Incidence and clinical outcomes of stent fractures on the basis of 6,555 patients and 16,482 drug-eluting stents from 4 centers. *JACC Cardiovasc Interv.* (2016) 9:1115–23. 10.1016/j.jcin.2016.02.025 27009464

[103] Lee S-H Park J-S Shin D-G Kim Y-J Hong G-R Kim W Frequency of stent fracture as a cause of coronary restenosis after sirolimus-eluting stent implantation. *Am J Card.* (2007) 100:627–30. 17697818 10.1016/j.amjcard.2007.03.073

[104] Yamada KP Koizumi T Yamaguchi H Kaneda H Bonneau HN Honda Y Serial angiographic and intravascular ultrasound analysis of late stent strut fracture of sirolimus-eluting stents in native coronary arteries. *Int J Cardiol.* (2008) 130:255–9. 10.1016/j.ijcard.2007.08.082 18096257

[105] Okamura T Matsuzaki M. Sirolimus-eluting stent fracture detection by three-dimensional optical coherence tomography. *Catheter Cardiovasc Interv.* (2012) 79:628–32. 10.1002/ccd.23268 21805594

[106] Hiltrop N De Cock D Ferdinande B Adriaenssens T. Detailed in vivo visualization of stent fracture causing focal restenosis using 3D reconstruction software for high-resolution optical coherence tomography images. *Eur Heart J Cardiovasc Imaging.* (2014) 15:714. 10.1093/ehjci/jet273 24408933

[107] Acar RD Bulut M Akcakoyun M. Are we aware of stent fracture? *Herz.* (2015) 40:417–22. 24154880 10.1007/s00059-013-3947-3

[108] Alexopoulos D Xanthopoulou I. Coronary stent fracture: how frequent it is? Does it matter? *Hellenic J Cardiol.* (2011) 52:1–5. 21292601

[109] Schochlow K Weissner M Blachutzik F Boeder NF Trobs M Lorenz L Coronary stent strut fractures: Classification, prevalence and clinical associations. *J Clin Med.* (2021) 10:1765. 10.3390/jcm10081765 33921606 PMC8072680

[110] Rhee TM Park KW Lee JM Lee MS Jeon KH Kang HJ Predictors and long-term clinical outcome of longitudinal stent deformation: Insights from pooled analysis of korean multicenter drug-eluting stent cohort. *Circ Cardiovasc Interv.* (2017) 10:e005518. 10.1161/CIRCINTERVENTIONS.117.005518 29146671

[111] Takebayashi H Mintz GS Carlier SG Kobayashi Y Fujii K Yasuda T Nonuniform strut distribution correlates with more neointimal hyperplasia after sirolimus-eluting stent implantation. *Circulation.* (2004) 110:3430–4. 10.1161/01.CIR.0000148371.53174.05 15557367

[112] Mehran R Dangas G Abizaid AS Mintz GS Lansky AJ Satler LF Angiographic patterns of in-stent restenosis: Classification and implications for long-term outcome. *Circulation.* (1999) 100:1872–8. 10.1161/01.cir.100.18.1872 10545431

[113] Alfonso F Perez-Vizcayno MJ Hernandez R Bethencourt A Marti V Lopez-Minguez JR A randomized comparison of sirolimus-eluting stent with balloon angioplasty in patients with in-stent restenosis: Results of the restenosis intrastent: Balloon angioplasty versus elective sirolimus-eluting stenting (RIBS-II) trial. *J Am Coll Cardiol.* (2006) 47:2152–60. 10.1016/j.jacc.2005.10.078 16750678

[114] Shlofmitz E Iantorno M Waksman R. Restenosis of drug-eluting stents: A new classification system based on disease mechanism to guide treatment and state-of-the-art review. *Circ Cardiovasc Interv.* (2019) 12:e007023. 10.1161/CIRCINTERVENTIONS.118.007023 31345066

[115] Goto K Zhao Z Matsumura M Dohi T Kobayashi N Kirtane AJ Mechanisms and patterns of intravascular ultrasound in-stent restenosis among bare metal stents and first- and second-generation drug-eluting stents. *Am J Cardiol.* (2015) 116:1351–7. 26341188 10.1016/j.amjcard.2015.07.058

[116] Jensen LO Vikman S Antonsen L Kosonen P Niemela M Christiansen EH Intravascular ultrasound assessment of minimum lumen area and intimal hyperplasia in in-stent restenosis after drug-eluting or bare-metal stent implantation. The Nordic Intravascular Ultrasound Study (NIVUS). *Cardiovasc Revasc Med.* (2017) 18:577–82. 29066343 10.1016/j.carrev.2017.05.010

[117] Her AY Shin ES. Current management of in-stent restenosis. *Korean Circ J.* (2018) 48:337–49. 29737639 10.4070/kcj.2018.0103 PMC5940640

[118] Byrne RA Joner M Tada T Kastrati A. Restenosis in bare metal and drug-eluting stents: Distinct mechanistic insights from histopathology and optical intravascular imaging. *Minerva Cardioangiol.* (2012) 60:473–89. 23018428

[119] Song L Mintz GS Yin D Yamamoto MH Chin CY Matsumura M Neoatherosclerosis assessed with optical coherence tomography in restenotic bare metal and first- and second-generation drug-eluting stents. *Int J Cardiovasc Imaging.* (2017) 33:1115–24. 10.1007/s10554-017-1106-2 28281026

[120] Ando H Suzuki A Sakurai S Kumagai S Kurita A Waseda K Tissue characteristics of neointima in late restenosis: Integrated backscatter intravascular ultrasound analysis for in-stent restenosis. *Heart Vessels.* (2017) 32:531–8. 10.1007/s00380-016-0903-1 27730297

[121] Uchida Y Ichimiya S Ishii H Oishi H Aoki T Miki Y Impact of coronary stent fracture on restenotic neointimal tissue characterization after drug-eluting stent implantation. *Int Heart J.* (2017) 58:861–7. 10.1536/ihj.16-571 29151488

[122] Gonzalo N Serruys PW Okamura T van Beusekom HM Garcia-Garcia HM van Soest G Optical coherence tomography patterns of stent restenosis. *Am Heart J.* (2009) 158:284–93. 19619707 10.1016/j.ahj.2009.06.004

[123] Yamamoto W Fujii K Otsuji S Takiuchi S Kakishita M Ibuki M Optical coherence tomography characteristics of in-stent restenosis after drug-eluting stent implantation: A novel classification and its clinical significance. *Heart Vessels.* (2020) 35:38–45. 10.1007/s00380-019-01461-7 31250131

[124] Imanaka T Fujii K Hao H Shibuya M Saita T Kawakami R Ex vivo assessment of neointimal characteristics after drug-eluting stent implantation: Optical coherence tomography and histopathology validation study. *Int J Cardiol.* (2016) 221:1043–7. 10.1016/j.ijcard.2016.07.110 27447812

[125] Xhepa E Byrne RA Rivero F Rroku A Cuesta J Ndrepepa G Qualitative and quantitative neointimal characterization by optical coherence tomography in patients presenting with in-stent restenosis. *Clin Res Cardiol.* (2019) 108:1059–68. 10.1007/s00392-019-01439-5 30783752

[126] Van Mieghem CA Cademartiri F Mollet NR Malagutti P Valgimigli M Meijboom WB Multislice spiral computed tomography for the evaluation of stent patency after left main coronary artery stenting: A comparison with conventional coronary angiography and intravascular ultrasound. *Circulation.* (2006) 114:645–53. 10.1161/CIRCULATIONAHA.105.608950 16894038

[127] Andreini D Pontone G Bartorelli AL Trabattoni D Mushtaq S Bertella E Comparison of feasibility and diagnostic accuracy of 64-slice multidetector computed tomographic coronary angiography versus invasive coronary angiography versus intravascular ultrasound for evaluation of in-stent restenosis. *Am J Cardiol.* (2009) 103:1349–58. 10.1016/j.amjcard.2009.01.343 19427427

[128] Veselka J Cadova P Adla T Zemanek D. Dual-source computed tomography angiography and intravascular ultrasound assessment of restenosis in patients after coronary stenting for bifurcation left main stenosis: A pilot study. *Arch Med Sci.* (2012) 8:455–61. 10.5114/aoms.2012.29220 22852000 PMC3400902

[129] Pang JH Kim D Beohar N Meyers SN Lloyd-Jones D Yaghmai V. Detection of stent fractures: A comparison of 64-slice CT, conventional cine-angiography, and intravascular ultrasonography. *Acad Radiol.* (2009) 16:412–7. 10.1016/j.acra.2008.10.010 19268852

[130] Li P Gai L. Coronary stent fracture detected by multidetector computed tomography. *Int J Cardiovasc Imaging.* (2010) 26:729–30. 20349340 10.1007/s10554-010-9624-1

[131] Kang SJ Cho YR Park GM Ahn JM Han SB Lee JY Predictors for functionally significant in-stent restenosis: An integrated analysis using coronary angiography, IVUS, and myocardial perfusion imaging. *JACC Cardiovasc Imaging.* (2013) 6:1183–90. 10.1016/j.jcmg.2013.09.006 24229771

[132] Neumann FJ Sousa-Uva M Ahlsson A Alfonso F Banning AP Benedetto U [2018 ESC/EACTS guidelines on myocardial revascularization. The Task Force on myocardial revascularization of the European society of cardiology (ESC) and EUROPEAN (EACTS)]. *G Ital Cardiol (Rome)* (2019) 20:1S–61S. 31379378 10.1714/3203.31801

[133] Stefanini GG Alfonso F Barbato E Byrne RA Capodanno D Colleran R Management of myocardial revascularisation failure: An expert consensus document of the EAPCI. *EuroIntervention.* (2020) 16:e875–90. 10.4244/EIJ-D-20-00487 32597391

[134] Lawton JS Tamis-Holland JE Bangalore S Bates ER Beckie TM Bischoff JM 2021 ACC/AHA/SCAI guideline for coronary artery revascularization: A report of the American college of cardiology/American heart association joint committee on clinical practice Guidelines. *Circulation* (2021):145:e4–17. 34882435 10.1161/CIR.0000000000001038

[135] Schiele F Meneveau N Seronde MF Deforet MF Gupta S Bassand JP. Predictors of event-free survival after repeat intracoronary procedure for in-stent restenosis; Study with angiographic and intravascular ultrasound imaging. *Eur Heart J.* (2000) 21:754–62. 10.1053/euhj.1999.1906 10739731

[136] Shlofmitz E Case BC Chen Y Chezar-Azerrad C Hashim H Garcia-Garcia HM Waksman in-stent restenosis classification: A mechanism-based approach to the treatment of restenosis. *Cardiovasc Revasc Med.* (2021) 33:62–7. 10.1016/j.carrev.2021.06.004 34247983

[137] Ferri LA Jabbour RJ Giannini F Benincasa S Ancona M Regazzoli D Safety and efficacy of rotational atherectomy for the treatment of undilatable underexpanded stents implanted in calcific lesions. *Catheter Cardiovasc Interv.* (2017) 90:E19–24. 10.1002/ccd.26836 27862848

[138] Hernandez-Enriquez M Campelo-Parada F Lhermusier T Bouisset F Roncalli J Elbaz M Long-term outcomes of rotational atherectomy of underexpanded stents. A single center experience. *J Interv Cardiol.* (2018) 31:465–70. 10.1111/joic.12491 29372576 PMC6099470

[139] Edes IF Ruzsa Z Szabo G Lux A Geller L Molnar L Rotational atherectomy of undilatable coronary stents: Stentablation, a clinical perspective and recommendation. *EuroIntervention.* (2016) 12:e632–5. 10.4244/EIJV12I5A103 27497363

[140] Latib A Takagi K Chizzola G Tobis J Ambrosini V Niccoli G Excimer laser lesion modification to expand non-dilatable stents: The ELLEMENT registry. *Cardiovasc Revasc Med.* (2014) 15:8–12. 10.1016/j.carrev.2013.10.005 24290659

[141] Blachutzik F Honton B Escaned J Hill JM Werner N Banning AP Safety and effectiveness of coronary intravascular lithotripsy in eccentric calcified coronary lesions: A patient-level pooled analysis from the Disrupt CAD I and CAD II Studies. *Clin Res Cardiol.* (2021) 110:228–36. 10.1007/s00392-020-01737-3 32948882 PMC7862504

[142] Mattesini A Nardi G Martellini A Sorini Dini C Hamiti B Stolcova M Intravascular imaging to guide lithotripsy in concentric and eccentric calcific coronary lesions. *Cardiovasc Revasc Med.* (2020) 21:1099–105. 10.1016/j.carrev.2020.04.016 32471713

[143] Alfonso F Bastante T Antuna P de la Cuerda F Cuesta J Garcia-Guimaraes M Coronary lithoplasty for the treatment of undilatable calcified de novo and in-stent restenosis lesions. *JACC Cardiovasc Interv.* (2019) 12:497–9. 30772288 10.1016/j.jcin.2018.12.025

[144] Hill JM Kereiakes DJ Shlofmitz RA Klein AJ Riley RF Price MJ Intravascular lithotripsy for treatment of severely calcified coronary artery disease. *J Am Coll Cardiol.* (2020) 76:2635–46. 33069849 10.1016/j.jacc.2020.09.603

[145] Szolc P Guzik B Wiewiorka L Niewiara L Kleczynski P Legutko J. Intravascular lithotripsy for the treatment of a heavily calcified recurrent in-stent restenosis in patient with chronic coronary syndrome. *Kardiol Pol.* (2021) 79:1159–60. 10.33963/KP.a2021.0079 34350971

[146] Ali ZA McEntegart M Hill JM Spratt JC. Intravascular lithotripsy for treatment of stent underexpansion secondary to severe coronary calcification. *Eur Heart J.* (2020) 41:485–6. 30462174 10.1093/eurheartj/ehy747

[147] Kalogeropoulos AS Karamasis GV Pavlidis AN Papadothomakos N Sakadakis E Vardas P Combined shockwave intravascular lithotripsy and ultrahigh-pressure balloon dilatation for the treatment of stent underexpansion secondary to severe coronary calcification. *Kardiol Pol.* (2021) 79:205–6. 10.33963/KP.15753 33463989

[148] Tovar Forero MN Van Mieghem NM Daemen J. Stent underexpansion due to heavy coronary calcification resistant to rotational atherectomy: A case for coronary lithoplasty? *Catheter Cardiovasc Interv.* (2020) 96:598–600. 10.1002/ccd.28641 31789483 PMC7540327

[149] Tovar Forero MN Wilschut J Van Mieghem NM Daemen J. Coronary lithoplasty: A novel treatment for stent underexpansion. *Eur Heart J.* (2019) 40:221. 10.1093/eurheartj/ehy593 30289452

[150] Brunner FJ Becher PM Waldeyer C Zengin-Sahm E Schnabel RB Clemmensen P Intravascular lithotripsy for the treatment of calcium-mediated coronary in-stent restenoses. *J Invasive Cardiol.* (2021) 33:E25–31. 33385983 10.25270/jic/20.00285

[151] Yeoh J Cottens D Cosgrove C Mallek K Strange J Anderson R Management of stent underexpansion using intravascular lithotripsy-defining the utility of a novel device. *Catheter Cardiovasc Interv.* (2021) 97:22–9. 10.1002/ccd.28715 31912981

[152] Wiens EJ Sklar JC Wei YH Aleem Q Minhas K. Real-world outcomes in treatment of highly calcified coronary lesions with intravascular shockwave lithotripsy. *Indian Heart J.* (2021) 73:653–5. 10.1016/j.ihj.2021.09.002 34627588 PMC8514397

[153] El Jattari H Holvoet W De Roeck F Cottens D Ungureanu C Bennett J Intracoronary lithotripsy in calcified coronary lesions: A multicenter observational study. *J Invasive Cardiol.* (2022) 34:E24–31. 34919529 10.25270/jic/21.00021

[154] Nikolakopoulos I Vemmou E Xenogiannis I Brilakis ES. Combined use of intravascular lithotripsy and brachytherapy: A new approach for the treatment of recurrent coronary in-stent restenosis. *Catheter Cardiovasc Interv.* (2021) 97:1402–6. 10.1002/ccd.29332 33031640

[155] Mousa MAA Bingen BO Amri IA Digiacomo S Karalis I Jukema JW Bail-out intravascular lithotripsy for the treatment of acutely underexpanded stents in heavily calcified coronary lesions: A case series. *Cardiovasc Revasc Med.* (2022) 40:189–94. 10.1016/j.carrev.2021.12.002 35063371

[156] Otsuka F Vorpahl M Nakano M Foerst J Newell JB Sakakura K Pathology of second-generation everolimus-eluting stents versus first-generation sirolimus– and paclitaxel-eluting stents in humans. *Circulation.* (2014) 129:211–23. 24163064 10.1161/CIRCULATIONAHA.113.001790 PMC3915802

[157] Inoue T Shite J Yoon J Shinke T Otake H Sawada T Optical coherence evaluation of everolimus-eluting stents 8 months after implantation. *Heart.* (2011) 97:1379–84. 21051456 10.1136/hrt.2010.204339

[158] Chen G Zrenner B Pyxaras SA. Combined rotational atherectomy and intravascular lithotripsy for the treatment of severely calcified in-stent neoatherosclerosis: A mini-review. *Cardiovasc Revasc Med.* (2019) 20:819–21. 10.1016/j.carrev.2018.10.007 30409500

[159] Chan KH Sia JE Tan HC. Intravascular lithotripsy for the treatment of severe calcific neointimal hyperplasia in a bare metal stent 17 years after implantation. *Coron Artery Dis.* (2021) 32:172–4. 10.1097/MCA.0000000000000905 32398575

[160] Salazar C Escaned J Tirado G Gonzalo N. Intravascular lithotripsy for recurrent restenosis caused by severe calcific neoatherosclerosis. *EuroIntervention.* (2020) 16:e351–2. 10.4244/EIJ-D-19-00268 31334702

[161] Giacoppo D Gargiulo G Aruta P Capranzano P Tamburino C Capodanno D. Treatment strategies for coronary in-stent restenosis: Systematic review and hierarchical Bayesian network meta-analysis of 24 randomised trials and 4880 patients. *BMJ.* (2015) 351:h5392. 10.1136/bmj.h5392 26537292 PMC4632210

[162] Pleva L Kukla P Kusnierova P Zapletalova J Hlinomaz O. Comparison of the efficacy of paclitaxel-eluting balloon catheters and everolimus-eluting stents in the treatment of coronary in-stent restenosis: The treatment of in-stent restenosis study. *Circ Cardiovasc Interv.* (2016) 9:e003316. 10.1161/CIRCINTERVENTIONS.115.003316 27069104

[163] Siontis GC Stefanini GG Mavridis D Siontis KC Alfonso F Perez-Vizcayno MJ Percutaneous coronary interventional strategies for treatment of in-stent restenosis: A network meta-analysis. *Lancet.* (2015) 386:655–64. 26334160 10.1016/S0140-6736(15)60657-2

[164] Lee JM Park J Kang J Jeon KH Jung JH Lee SE Comparison among drug-eluting balloon, drug-eluting stent, and plain balloon angioplasty for the treatment of in-stent restenosis: A network meta-analysis of 11 randomized, controlled trials. *JACC Cardiovasc Interv.* (2015) 8:382–94. 10.1016/j.jcin.2014.09.023 25703886

[165] Giacoppo D Alfonso F Xu B Claessen B Adriaenssens T Jensen C Paclitaxel-coated balloon angioplasty vs. drug-eluting stenting for the treatment of coronary in-stent restenosis: A comprehensive, collaborative, individual patient data meta-analysis of 10 randomized clinical trials (DAEDALUS study). *Eur Heart J.* (2020) 41:3715–28. 31511862 10.1093/eurheartj/ehz594 PMC7706792

[166] Tada T Kadota K Hosogi S Miyake K Ohya M Amano H Association between tissue characteristics assessed with optical coherence tomography and mid-term results after percutaneous coronary intervention for in-stent restenosis lesions: A comparison between balloon angioplasty, paclitaxel-coated balloon dilatation, and drug-eluting stent implantation. *Eur Heart J Cardiovasc Imaging.* (2015) 16:1101–11. 10.1093/ehjci/jev031 25762559

[167] Gonzalo N Salazar CH Perez-Vizcayno MJ Gomez-Polo JC Jimenez-Quevedo P Jimenez-Valero S Influence of neoatherosclerosis on prognosis and treatment response in patients with in-stent restenosis. *Rev Esp Cardiol (Engl Ed).* (2021) 74:427–35. 10.1016/j.rec.2020.03.005 32439297

[168] Adriaenssens T Dens J Ughi G Bennett J Dubois C Sinnaeve P Optical coherence tomography study of healing characteristics of paclitaxel-eluting balloons vs. everolimus-eluting stents for in-stent restenosis: The SEDUCE (safety and efficacy of a drug eluting balloon in coronary artery restenosis) randomised clinical trial. *EuroIntervention.* (2014) 10:439–48. 10.4244/EIJV10I4A77 25138182

[169] Alfonso F Perez-Vizcayno MJ Garcia Del Blanco B Otaegui I Masotti M Zueco J Long-term results of everolimus-eluting stents versus drug-eluting balloons in patients with bare-metal in-stent restenosis: 3-year follow-up of the RIBS v clinical trial. *JACC Cardiovasc Interv.* (2016) 9:1246–55. 10.1016/j.jcin.2016.03.037 27339840

[170] Rivero F Bastante T Cuesta J Benedicto A Restrepo JA Alfonso F. Treatment of in-stent restenosis with bioresorbable vascular scaffolds: Optical coherence tomography insights. *Can J Cardiol.* (2015) 31:255–9. 25660152 10.1016/j.cjca.2014.11.017

[171] Alfonso F Cuesta J Perez-Vizcayno MJ Garcia Del Blanco B Rumoroso JR Bosa F Bioresorbable vascular scaffolds for patients with in-stent restenosis: The RIBS VI study. *JACC Cardiovasc Interv.* (2017) 10:1841–51. 28866036 10.1016/j.jcin.2017.06.064

[172] Picard F Avram R Marquis-Gravel G Tadros VX Ly HQ de Hemptinne Q Bioresorbable vascular scaffold to treat in-stent restenosis: Single-center experience. *J Interv Cardiol.* (2017) 30:558–63. 28786151 10.1111/joic.12420

[173] Moscarella E Tanaka A Ielasi A Cortese B Coscarelli S De Angelis MC Bioresorbable vascular scaffold versus everolimus-eluting stents or drug eluting balloon for the treatment of coronary in-stent restenosis: 1-year follow-up of a propensity score matching comparison (the BIORESOLVE-ISR Study). *Catheter Cardiovasc Interv.* (2018) 92:668–77. 29356269 10.1002/ccd.27473

[174] Negi SI Torguson R Gai J Kiramijyan S Koifman E Chan R Intracoronary brachytherapy for recurrent drug-eluting stent failure. *JACC Cardiovasc Interv.* (2016) 9:1259–65. 27339842 10.1016/j.jcin.2016.03.018

[175] Varghese MJ Bhatheja S Baber U Kezbor S Chincholi A Chamaria S Intravascular brachytherapy for the management of repeated multimetal-layered drug-eluting coronary stent restenosis. *Circ Cardiovasc Interv.* (2018) 11:e006832. 10.1161/CIRCINTERVENTIONS.118.006832 30354630

[176] Yabushita H Kawamoto H Fujino Y Tahara S Horikoshi T Tada M Clinical outcomes of drug-eluting balloon for in-stent restenosis based on the number of metallic layers. *Circ Cardiovasc Interv.* (2018) 11:e005935. 10.1161/CIRCINTERVENTIONS.117.005935 30354780

[177] Fujii K Mintz GS Kobayashi Y Carlier SG Takebayashi H Yasuda T Contribution of stent underexpansion to recurrence after sirolimus-eluting stent implantation for in-stent restenosis. *Circulation.* (2004) 109:1085–8. 10.1161/01.CIR.0000121327.67756.19 14993129

[178] Yin D Mintz GS Song L Chen Z Lee T Kirtane AJ In-stent restenosis characteristics and repeat stenting underexpansion: Insights from optical coherence tomography. *EuroIntervention.* (2020) 16:e335–43. 10.4244/EIJ-D-18-01191 31403461

[179] Sakamoto Y Yamawaki M Araki M Kobayashi N Mori S Tsutsumi M Comparison of 12-month angiographic outcomes between repeat drug-eluting stent implantation and drug-coated balloon treatment for restenotic lesion caused by stent fracture. *Heart Vessels.* (2019) 34:1589–94. 10.1007/s00380-019-01398-x 30963303

[180] Popma JJ Tiroch K Almonacid A Cohen S Kandzari DE Leon MB. A qualitative and quantitative angiographic analysis of stent fracture late following sirolimus-eluting stent implantation. *Am J Cardiol.* (2009) 103:923–9. 10.1016/j.amjcard.2008.12.022 19327417

[181] Shlofmitz E Torguson R Mintz GS Zhang C Sharp A Hodgson JM The IMPact on revascularization outcomes of intravascular ultrasound-guided treatment of complex lesions and economic impact (IMPROVE) trial: Study design and rationale. *Am Heart J.* (2020) 228:65–71. 10.1016/j.ahj.2020.08.002 32866927

[182] Alberti A Giudice P Gelera A Stefanini L Priest V Simmonds M Understanding the economic impact of intravascular ultrasound (IVUS). *Eur J Health Econ.* (2016) 17:185–93. 25669755 10.1007/s10198-015-0670-4

[183] Waters KR Bautista R Zelenka R Masters D Reynolds JS Nelson S Development of a high-definition intravascular ultrasound imaging system and catheter. In: *Proceedings of the 2011 IEEE International Ultrasonics Symposium; 2011 18-21 Oct*, Orlando, FL: IEEE. (2011).

[184] Garcia-Guimaraes M Antuna P De la Cuerda F Maruri-Sanchez R Cuesta J Bastante T High-definition IVUS versus OCT to assess coronary artery disease and results of stent implantation. *JACC Cardiovasc Imaging.* (2020) 13:519–21. 10.1016/j.jcmg.2019.08.019 31607666

[185] Kim TS Park H-S Jang S-J Song JW Cho HS Kim S Single cardiac cycle three-dimensional intracoronary optical coherence tomography. *Biomed Opt Express.* (2016) 7:4847–58. 28018710 10.1364/BOE.7.004847 PMC5175536

[186] Saito Y Kitahara H Okuya Y Nakayama T Fujimoto Y Kobayashi Y. Novel predictor of target vessel revascularization after coronary stent implantation: Intraluminal intensity of blood speckle on intravascular ultrasound. *Catheter Cardiovasc Interv.* (2019) 93:604–10. 10.1002/ccd.27859 30269414

