# Supplementary material for: Intravascular imaging in coronary stent restenosis: Prevention, characterization, and management
Source: Front Cardiovasc Med. 2022 Aug 9;9:843734. doi: 10.3389/fcvm.2022.843734 (PMC9395642; doi:10.3389/fcvm.2022.843734)
Supplement: Supplementary file 1 [file Image_1.pdf]

Supplementary Material

1 Supplementary Figures and Tables

1.1 Supplementary Figures

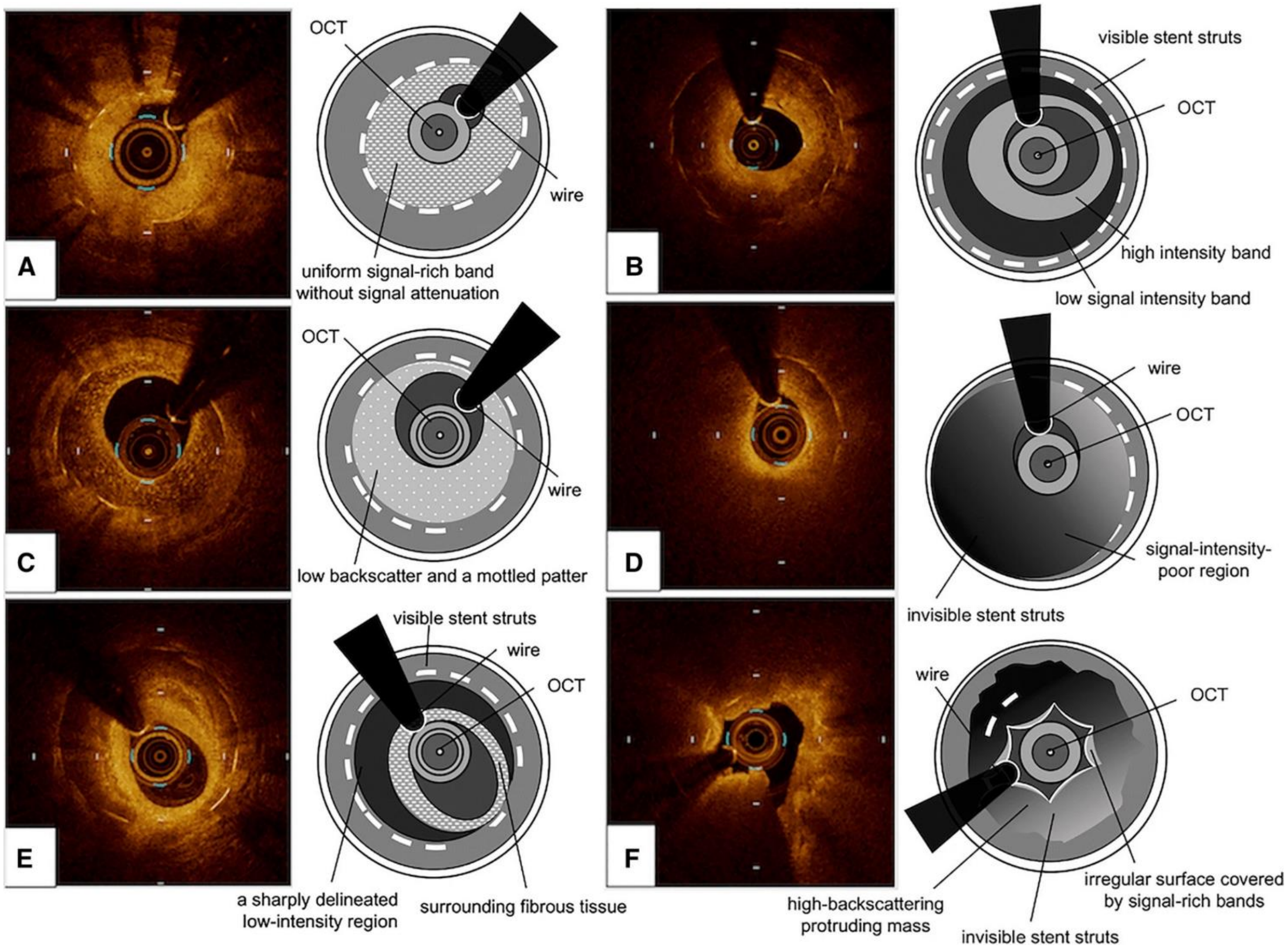

Supplementary Figure 1. Novel OCT-based classification of ISR after DES implantation, proposed by Yamamoto et al. A) Type I “Homogeneous high-intensity tissue”. B) Type II “Heterogeneous tissue with signal attenuation”. C) Type III “Speckled heterogeneous tissue”. D) Type IV “Heterogeneous tissue containing poorly delineated region with invisible strut”. E) Type V “Heterogeneous tissue containing sharply delineated low-intensity region”. F) Type VI “Bright protruding tissue with an irregular surface”. Reproduced with permission from (121).
